# Electrosynthesis of Atomically Precise Au Nanoclusters

**DOI:** 10.1002/advs.202414057

**Published:** 2025-03-13

**Authors:** Jing Dong, Yawei Li, Yu Ding, Hai‐Feng Su, Xiaoqin Cui, Yu‐Xin Wang, Huan Li

**Affiliations:** ^1^ Institute of Crystalline Materials Shanxi University Taiyuan Shanxi 030006 China; ^2^ School of Chemistry and Chemical Engineering Shanxi University Taiyuan Shanxi 030006 China; ^3^ Department of Chemistry College of Chemistry and Chemical Engineering Xiamen University Xiamen 361005 P. R. China

**Keywords:** Au nanoclusters, electrosynthesis, pulsed electrolysis

## Abstract

Innovation in synthesis methodologies is crucial for advancing the discovery of new materials. This work reports the electrosynthesis of a [Au_13_(4‐*^t^* BuPhC≡C)_2_(Dppe)_5_]Cl_3_ nanocluster (**Au_13_** **NC)** protected by alkynyl and phosphine ligands. From simple precursor, HAuCl_4_ and ligands, the whole synthesis is driven by a constant potential in single electrolytic cell. X‐ray crystallography determines its total structure. Control experiments, cyclic voltammetry, Proton Nuclear Magnetic Resonance (^1^H NMR), gas chromatography, and other characterizations demonstrate that a critical tetranuclear Au(I) complex defines the electrochemical redox behavior of the reaction solution. The critical role of a base (e.g., triethylamine) is to suppress the hydrogen evolution reaction at the cathode, paving the way for the reduction of Au ions. To resolve the problem of over‐reduction and deposition of Au on the cathode, pulsed electrolysis, which is specific to electrosynthesis is employed. It significantly improves the reaction rate and the isolated yield of **Au_13_**. To extend the application scope, another four NCs protected by different ligands, [Au_13_(4‐FPhC≡C)_2_(Dppe)_5_]Cl_3_, [Au_8_(2‐CF_3_PhC≡C)_2_(Dppp)_4_](PF_6_)_2_, [Au_11_(Dppp)_5_]Cl_3_, and [Au_8_(SC_2_H_4_Ph)_2_(Dppp)_4_]Cl_2_ are synthesized electrochemically, demonstrating the versatility of the strategy.

## Graphical abstract

In this work, atomically precise Au nanoclusters are fabricated by electrochemical method. The synthesis mechanism is explored. Simply by adjusting the potential waveform, the yield of Au nanoclusters is significantly enhanced, highlighting its technical superiority. To expand the application scope, another four Au nanoclusters are produced by replacing certain ingredients, demonstrating the versatility of the strategy.

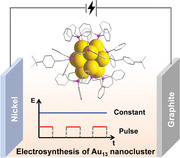

## 1 Introduction

Electrosynthesis uses electrical energy to drive chemical reactions, avoiding using some harsh oxidizing and reducing reagents.^[^ ^1^, ^2^ ^]^ The magnitude and waveform of the electrical potential can be precisely tuned to adjust the reaction rate and selectivity. The merges of electrochemistry with organocatalysis, flow technique, and biochemistry open an even broader application prospect. Thanks to the advantages, recent decades have witnessed an ongoing renaissance of the field. A wide range of organic compounds that would otherwise be difficult to obtain have been electrochemically synthesized.^[^ ^3^, ^4^, ^5^, ^6^, ^7^, ^8^, ^9^, ^10^, ^11^, ^12^, ^13^, ^14^ ^]^ For metal‐based materials, electrosynthesis has also proved to be a reliable and versatile strategy. A plethora of metal nanoparticles (nanoclusters),^[^ ^15^, ^16^, ^17^, ^18^ ^]^ alloy structures,^[^ ^19^, ^20^ ^]^ metal oxides,^[^ ^21^, ^22^ ^]^ and semiconductors^[^ ^23^, ^24^ ^]^ with various sizes and morphologies have been fabricated. Taking gold as example, the wafer‐size foil,^[^ ^25^ ^]^ nanopore arrays,^[^ ^26^ ^]^ nanowire,^[^ ^27^ ^]^ and nanoparticles^[^ ^28^, ^29^ ^]^ can all be acquired by electrochemical method. Abundant results have convincingly demonstrated the capability of electrosynthesis in a controllable manner at the nanoscale. Transmission Electron Microscope (TEM), Atomic Force Microscope (AFM), Electrospray Ionization Mass Spectrometry (ESI‐MS), and other techniques that have subnanometer to nanometer resolution are usually adequate for their morphology and composition characterization.^[^ ^30^ ^]^ Electrochemical methods have been proven to be effective for metal nanoclusters synthesis.^[^ ^31^, ^32^ ^]^ Till now, however, single crystals of Au nanoclusters via electrosynthesis have not been isolated yet.

Au nanoclusters (NCs) keep attracting the community's attention owing to their unique physicochemical properties, such as multiple absorption bands, distinct photoluminescence, and well‐defined catalytic sites.^[^ ^33^, ^34^, ^35^, ^36^, ^37^, ^38^ ^]^ To gain a fundamental insight into their structure–property relationship, ligand‐protected Au NCs whose structures can be determined by single crystal X‐ray diffraction are regarded as ideal research models, because their metal cores, peripheral ligands, and stacking modes can all be precisely mapped out. To synthesize them, chemists have mostly relied on solution phase synthesis using a reducing agent (typically, NaBH_4_). The method has given birth to a wealth of Au NCs with different nuclearities and ligands.^[^ ^39^, ^40^, ^41^, ^42^, ^43^, ^44^, ^45^, ^46^, ^47^, ^48^ ^]^ The well‐researched **Au_25_** NC, for example, was afforded by reducing HAuCl_4_ with NaBH_4_ under the protection of a 2‐phenylethanethiol ligand.^[^ ^49^ ^]^ The discoveries of other brilliant methods, such as photochemical method,^[^ ^50^, ^51^ ^]^ solid‐state synthesis,^[^ ^52^ ^]^ etc., have not only provided alternative pathways toward NC, but also shed new light on the formation mechanism.^[^ ^53^, ^54^, ^55^, ^56^ ^]^

In this work, an Au NC with atomic precision is synthesized in an electrolytic cell for the first time. From a simple precursor, HAuCl_4_ to a sophisticated [Au_13_(4‐*^t^* BuPhC≡C)_2_(Dppe)_5_]Cl_3_ (**Au_13_**), the whole synthesis is driven by a constant potential. The electrochemical redox behavior study, the capture of a critical tetranuclear Au(I) intermediate and the elucidation of the role of amine help to clarify the formation mechanism of **Au_13_** under electrochemical conditions. To resolve the problem of over‐reduction and deposition of Au on the cathode, pulsed electrolysis is employed, which significantly improves the reaction rate and the isolated yield. To expand the application scope of the strategy, another four Au NCs protected by different ligands including ([Au_13_(4‐FPhC≡C)_2_(Dppe)_5_]Cl_3_, [Au_8_(SC_2_H_4_Ph)_2_(Dppp)_4_]Cl_2_, [Au_11_(Dppp)_5_]Cl_3_, [Au_8_(2‐CF_3_PhC≡C)_2_(Dppp)_4_](PF_6_)_2_ are also successfully synthesized.

## 2 Results and Discussion

### 2.1 Electrosynthesis of Au_13_ Nanocluster

**Figure 1:**
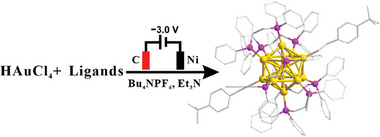
Illustration of the electrosynthesis of [Au_13_(4‐*^t^* BuPhC≡C)_2_(Dppe)_5_]Cl_3_ (**Au_13_)**. Ligands: 4‐*tert*‐butylphenylacetylene (TBA) and 1,2‐bis‐ (diphenylphosphino)ethane (Dppe). Anode: graphite. Cathode: Ni electrode. Color code: yellow, Au; pink, P; gray, C.

The electrosynthesis of **Au_13_** was conducted in a single electrolytic cell where a graphite electrode (ϕ 0.6 × 0.75 cm) served as anode (**Figure** **1**). Various materials were evaluated for the cathode, including glassy carbon, Ni, Pt, and graphite electrodes, etc. Based on cost‐effectiveness and practical results, Ni plate was employed in most cases. HAuCl_4_, dimethyl sulfide (Me_2_S) and the ligands (4‐*tert*‐butylphenylacetylene (TBA) and 1,2‐bis‐ (diphenylphosphino)ethane (Dppe)) were successively dissolved in a mixture of dichloromethane and ethanol, followed by the addition of triethylamine (Et_3_N). Me_2_S was employed reduce Au^3+^ to Au^+^, which a common procedure in the synthesis of Au NCs.^[^ ^41^, ^47^ ^]^ Tetrabutylammonium hexafluorophosphate (Bu_4_NPF_6_, 0.1 m) was employed to increase the conductivity (Figure 1). After a constant potential of –3 V was applied, the color of the solution gradually changed from colorless to brown‐red in a few hours (Figures S1 and S2, Supporting Information). The resulting dark transparent solution was centrifuged and concentrated for the following layering process, which yielded red block‐shaped crystals in a week. The single crystal was then subjected to characterization by X‐ray diffraction. Notably, further aging the as‐prepared reaction solution did not result in deterioration, indicating the acceptable stability of **Au_13_** under reaction conditions (Figures S3 and S4, Supporting Information). Furthermore, the elemental analysis unveiled negligible Ni^2+^ ions in the resulting solution, likely due to the fact that the Ni electrode, operating as the cathode, exhibited a much reduced tendency to dissolve.

**Figure 2:**
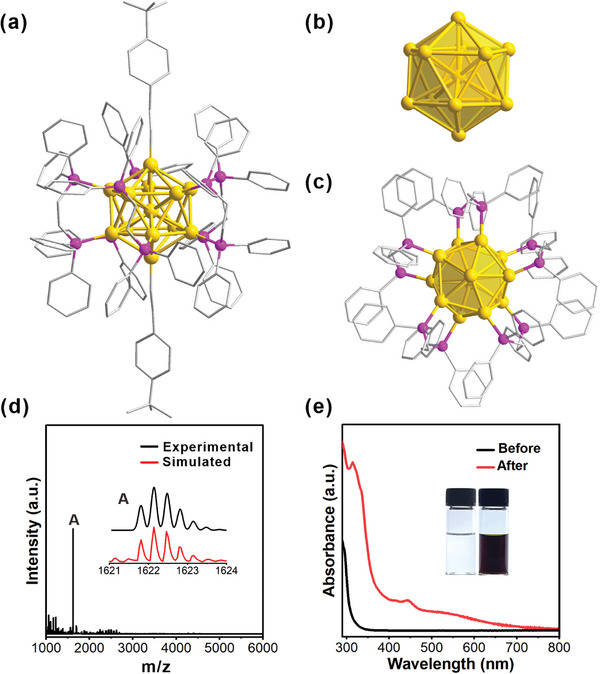
Total structure a) and core structure b) of **Au_13_**. c) Distribution and coordination patterns of phosphine ligands. d) Mass spectrum of **Au_13_**. Inset: experimental (black trace) and simulated (red trace) isotopic patterns of the molecular ion peak for [Au_13_(4‐*^t^* BuPhC≡CR)_2_(Dppe)_5_]Cl_3_. e) UV−vis absorption spectra of the **Au_13_** solution before (black line) and after 12 h electrolysis (red line). The inset is photographs of the vessel before (left) and after (right) electrolysis.

The crystal structure of **Au_13_** is shown in **Figure** **2**a. It has a centered icosahedron core composed of thirteen Au atoms (Figure 2b). Two alkynyl ligands are σ‐coordinated with Au of the two diagonal apexes of the icosahedron (Figure 2a). Five Dppe form a donut‐like belt covering the rest of the surface of the icosahedron (Figure 2c). The ESI mass spectrum of **Au_13_** gives a set of signals at around m/z 1622.37, agreeing with the calculated isotope pattern (Figure 2d). The presence of Cl^−^ counterions in the structure was also confirmed by ion chromatography (Figure S5, Supporting Information). According to the superatom theory, **Au_13_** has 8 free valence electrons (13_Au_−2_alkyne_−3_Cl_^−^ = 8e), the same with the previously reported **Au_13_** NCs mediated by NaBH_4_ (Figure S6, Supporting Information).^[^ ^57^ ^]^ On the other side, the weakening of the stretching mode of ν(≡C−H) at 3300 cm^−1^ in the infrared spectrum (IR) of **Au_13_** indicates the bonding of TBA with Au (Figure S7, Supporting Information). The UV–vis spectrum of **Au_13_** shows absorptions at 314, 446, and 545 nm (Figure 2e).^[^ ^57^ ^]^ The optical bandgap is calculated to be 1.49 eV (Figure S8, Supporting Information).^[^ ^58^ ^]^ The solution of **Au_13_** was also analyzed by square wave voltammetry (SWV, Figure S9, Supporting Information), from which the Highest Occupied Molecular Orbital ‐ Lowest Unoccupied Molecular Orbital gap (HOMO–LUMO gap) was estimated to be 1.38 eV.^[^ ^59^, ^60^ ^]^ Both results are in agreement.

### 2.2 Synthesis Mechanism Study

**Figure 3:**
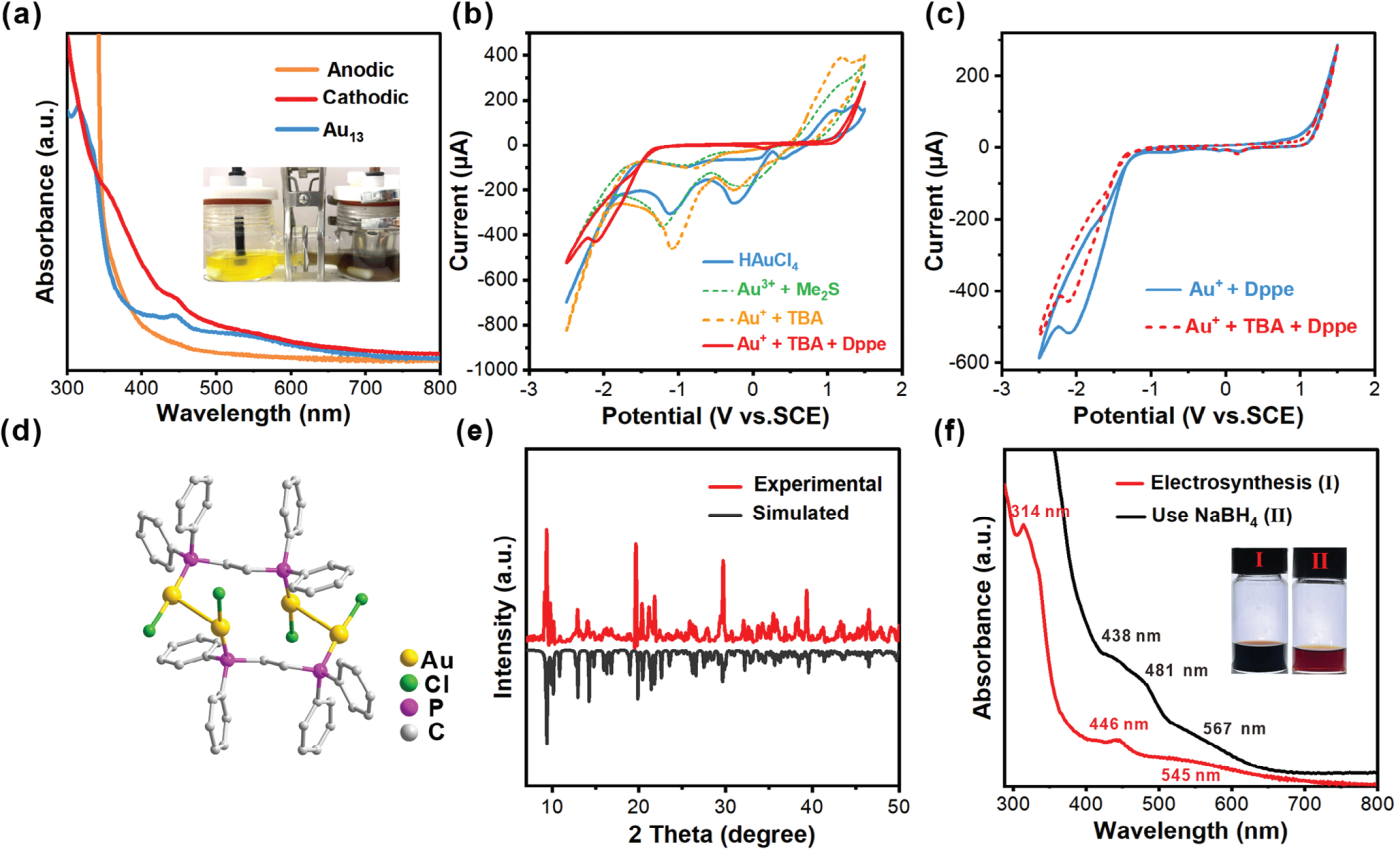
a) UV−vis spectra of the solutions from divided cell. The spectrum of **Au_13_** is included for comparison. Insert: the photograph of the reaction vessel after 12 h electrosynthesis. The right part of the vessel is the cathodic compartment. b) Cyclic voltammograms (CV) of the solution at different feeding stages (scan rate = 100 mV s^−1^). Me_2_S is dimethyl sulfide, TBA is 4‐*tert*‐butylphenylacetylene and Dppe is 1,2‐bis‐ (diphenylphosphino)ethane). c) The CV comparison of the solutions containing different components. They show evident resemblance. d) The crystal structure of Au_4_(Dppe)_2_Cl_4_. e) The powder XRD patterns of the dried reaction solution (before electrolysis, red line) and the one simulated from the single crystal of Au_4_(Dppe)_2_Cl_4_ (black line). f) UV–vis absorption spectra of Au nanoclusters synthesized by electrosynthesis (red line) and by NaBH_4_ (black line).

Since conventional synthesis of **Au_13_** NC required a reducing reagent, it was speculated **Au_13_** may form at the cathode which reduced the Au precursor by supplying electrons. In a control experiment, the same reactants were charged into both compartments of a divided cell separated by fiberglass membrane. Au species was reduced only in the cathodic compartment, as expected (**Figure** **3**a, inset). Then, the redox behavior at different feeding stages was studied by cyclic voltammetry (CV). As shown in Figure 3b, the solution of HAuCl_4_ shows two reduction peaks at −0.15 and −1.16 V (vs SCE), corresponding to Au(III)→Au(I) and Au(I) →Au(0) conversions.^[^ ^61^, ^62^ ^]^ The addition of Me_2_S and TBA did not result in significant change. The situation was completely altered when Dppe was introduced. Neither of the above peaks was observed. A more negative reduction peak at −2.1 V emerged. Such a negative shift may arise from the coordination of Dppe with Au. To verify this, only Dppe was used as ligand. The resulting CV in Figure 3c shows obvious resemblance in terms of both potential and shape of the peak.

Next, to capture the potential Au−Dppe complex that may be responsible for such dramatic change, the reaction solution (before electrolysis) was treated by layering. Colorless single crystals were collected in high yield (Figure S10, Supporting Information). X‐ray crystallography determined the crystal structure of this tetranuclear Au(I) complex to be Au_4_(Dppe)_2_Cl_4_ (Figure 3d). It has two Au atoms being bridged by one Dppe and each Au atom is coordinated with a Cl atom to form Au_2_(Dppe)Cl_2_. And Au_4_(Dppe)_2_Cl_4_ is composed of two Au_2_(Dppe)Cl_2_ parts, forming a centrosymmetric dimer through aurophilic interaction.^[^ ^63^ ^]^ The same solution that afforded Au_4_(Dppe)_2_Cl_4_ was also dried and subjected to powder XRD analysis (Figure 3e). The resulting pattern fits the simulation result from the crystal structure of Au_4_(Dppe)_2_Cl_4_. This means that Au_4_(Dppe)_2_Cl_4_ is likely the dominant Au related species in solution, which dictates its redox behavior. Because the peak potential was −2.1 V. To ensure that the cathodic reduction occurred, a more negative potential was required. Although the reaction proceeded at −2.5 V, the progress was sluggish. In practice, a potential of −3 V was applied effectively (Figures S11 and S12, Supporting Information).

During the course of electrosynthesis, the reaction solution was subjected to CV analysis at intervals. The combined results in Figure S13 (Supporting Information) show that the reduction peak current (i*_p_*) gradually decreased, indicating Au_4_(Dppe)_2_Cl_4_ was slowly consumed as the reaction went on. Eventually, the peak at −2.1 V disappeared, and the solution turned brown red. The CV curve, however, shows no corresponding oxidation peak in anodic scan, implying an irreversible electrode reaction (Figure 3c). To confirm that, CV scans at varying rates were conducted (30–250 mV s⁻¹, Figure S14, Supporting Information). The results showed a linear relationship between peak potential E*_p_* and logυ (*υ* is scan rate), meeting the Laviron equation (Figure S15, Supporting Information).^[^ ^64^ ^]^ Additionally, the reduction peak current *I_p_* plotted against υ^1/2^ followed the Randles–Sevcik equation,^[^ ^65^, ^66^ ^]^ indicating diffusion‐limited kinetics (Figure S16, Supporting Information).

**Figure 4:**
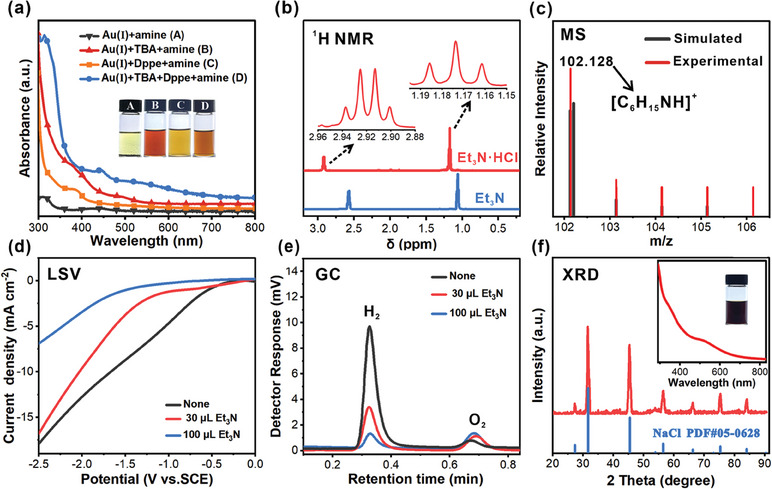
a) UV−vis spectra of the reaction solution after electrolysis with different reactants. Inset: corresponding photographs. b) ^1^H NMR spectra of reaction solution after electrosynthesis using HAuCl_4_ and Et_3_N (red line) and the spectrum of Et_3_N (blue line). c) High‐resolution mass spectrum of the reaction solution the same as that in (b). d) Linear sweep voltammetry (LSV) curves of reaction solution in the presence of different amounts of Et_3_N. Scan rate, *υ* = 5 mV s^−1^. Working electrode: Ni plate; counter electrode: graphite; reference electrode: saturated calomel electrode. e) Gas chromatography (GC) analysis: the signal response of H_2_ during electrosynthesis with different amount of Et_3_N. f) XRD pattern of the dried reaction solution using NaOH as a base. Inset: UV−vis spectrum and the photograph of vessel after reaction.

When ligands were not present, Au ions still got reduced, but the products were black precipitate (Au nanoparticles). When only alkyne was used as ligand, no stable NC colloidal solution was formed and cathode deposition of Au was evident. The reaction progress was sluggish when using only Dppe as ligand (**Figure** **4**a). No single crystal of Au NC was collected after work‐up in both cases. To explore the difference between the traditional and electrosynthesis, NaBH_4_ was employed using the same recipe without applying electric potential. The colors of the resulting solutions and the UV–vis absorption spectra showed obvious differences. The one using NaBH_4_ showed peaks at 438, 481, and 567 nm, quite different from that by electrosynthesis (Figure 3f). This may indicate different product structures. Through our observation, the reaction solution immediately turned from colorless to brown upon the introduction of NaBH_4_, while for electrosynthesis, the color transition was slow (typically hours before an obvious color change can be observed). The difference in reduction kinetics could be responsible for this, although the precise mechanism requires future thorough investigation. Curiously, without Et_3_N, the reaction did not proceed, either (Figure S17a, Supporting Information). To figure it out, different characterizations were performed. ^1^H NMR spectrum of the resulting solution shows the same H count and multiplicity as that of Et_3_N, although the signals shift to downfield (Figure 4b). This was empirically attributed to protonation of Et_3_N (Et_3_NH^+^). It was then confirmed by the mass spectroscopy by showing a signal at m/z 102.128 (calc. m/z 102.199, Figure 4c). The function of Et_3_N was to capture the protons which were likely from HAuCl_4_, 4‐*tert*‐butylphenylacetylene or protons produced by oxidation of solvents.

The ensuing question is why a relatively acidic environment suppressed the formation of **Au_13_**. Our speculation was that H^+^ reduction (hydrogen evolution reaction, HER) at the cathode may be a highly competitive reaction, since the reduction potential of Au(I) species was more negative than the reduction of H^+^. As shown in the linear sweep voltammetry (LSV, Figure 4d), in the absence of Et_3_N, the current of HER became evident at about −0.5 V, significantly more positive than that required for the synthesis of **Au_13_**. On the other hand, adding Et_3_N greatly suppressed the HER. For example, at −1.5 V, the current density was −8.64 mA cm^−2^ without Et_3_N. When Et_3_N was increased from 30 to 100 µL, the current density decreased markedly to −3.77 and −0.99 mA cm^−2^, respectively. The gas phase of the reaction vessel was also analyzed by gas chromatography equipped with thermal conductivity detector (TCD, Figure 4e). The sharp peak at retention time of 0.33 min was assigned to H_2_. In the absence of Et_3_N, H_2_ concentration was12244 ppm. The values dropped to 4567 and 1332 ppm when 30 and 100 µL Et_3_N were fed, respectively (Figure 4e). HER was effectively inhibited by Et_3_N, paving the way for the reduction of Au species. Furthermore, the more amine was used, the faster the reaction went, which is illustrated in Figure S17 (Supporting Information). With this information, we assumed that another base may also be effective. When NaOH was used instead of Et_3_N, the same **Au_13_** was produced (Figure 4f, inset). Powder XRD of the resulting dried solution showed NaCl was the product, indicating that NaOH was neutralized (Figure 4f). In a separate experiment, when HCl solution was deliberately charged into the vessel during the electrosynthesis of **Au_13_**, the formation of **Au_13_** ceased. And introducing NaOH restarted the progress (Figure S18, Supporting Information), further confirming our speculation.

### 2.3 Pulsed Potential Synthesis

**Figure 5:**
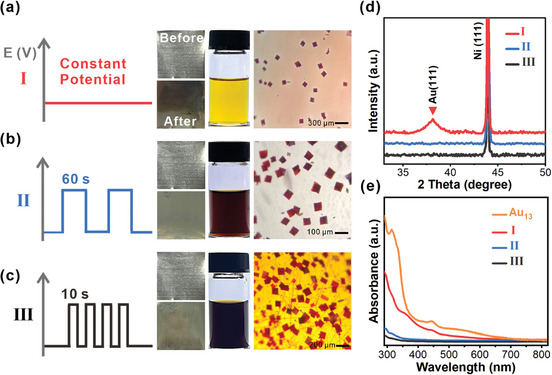
a) (I) represents the results obtained under constant potential. The photo on the left is the Ni electrode before and after electrosynthesis. The one in the middle is the reaction vessel after 4 h of electrosynthesis. The one on the right is the single crystal of **Au_13_**. (b) and (c) are corresponding results under pulsed potentials. Pulse width is 60 s for (II) and 10 seconds for (III). d) XRD patterns of the used Ni electrodes. e) UV–vis spectra of the solutions formed by rinsing the used Ni electrodes with CH_2_Cl_2._

Unfortunately, black deposit on the Ni electrode was always seen after electrosynthesis under constant potential. The isolated yield of **Au_13_** was relatively low (less than 13%, **Figure** **5**a, (I)). X‐ray diffraction pattern of the used Ni electrode showed an X‐ray diffraction peak at 38.2°, resulting from the face‐centered cubic Au (111) plane. A portion of Au(I) was reduced to Au nanoparticles (Figure 5d, (I)). Also, after rinsing the used Ni. electrode with CH_2_Cl_2_, the collected solution showed similar characteristic absorptions with that of **Au_13_** (Figure 5e, (I)). These results indicated under constant potential, Au(I) species tended to be over reduced and deposited on the electrode. To alleviate the problem, we resorted to pulsed potential electrolysis, which occasionally showed dramatic effect in organic electrosynthesis.^[^ ^67^, ^68^ ^]^ To our delight, this technique turned out to be very effective. When the pulse width was set to 60 s, (on for 60 s, off for 60 s), the reaction proceeded obviously faster (Figure S19, Supporting Information), and the isolated yield of **Au_13_** increased to an average of 28% (Figure 5b, (II)). Shortening the pulse width to 10 s further accelerated the reaction. Even more **Au_13_** crystals were collected after workup (isolated yield up to 36%, Figure 5c, (III)). Further shortening of the pulse interval did not result in significant changes. Both the XRD patterns and spectral features showed no sign of over‐reduction or NC deposition (Figures 5d,e.) It is deduced that the Au(0) species deposited on the cathode may inhibit the continuous reduction of Au(I) species under constant potential. The pulsed potential allows the reduced species to leave the cathodic region to avoid over‐reduction. Although the underlying mechanism is yet to be fully verified, simply adjusting the potential waveform can increase the yield of Au NCs, demonstrating the technical advantage of electrosynthesis that is difficult to achieve through conventional synthesis methods.

### 2.4 Application Scope of Electrosynthesis

**Figure 6:**
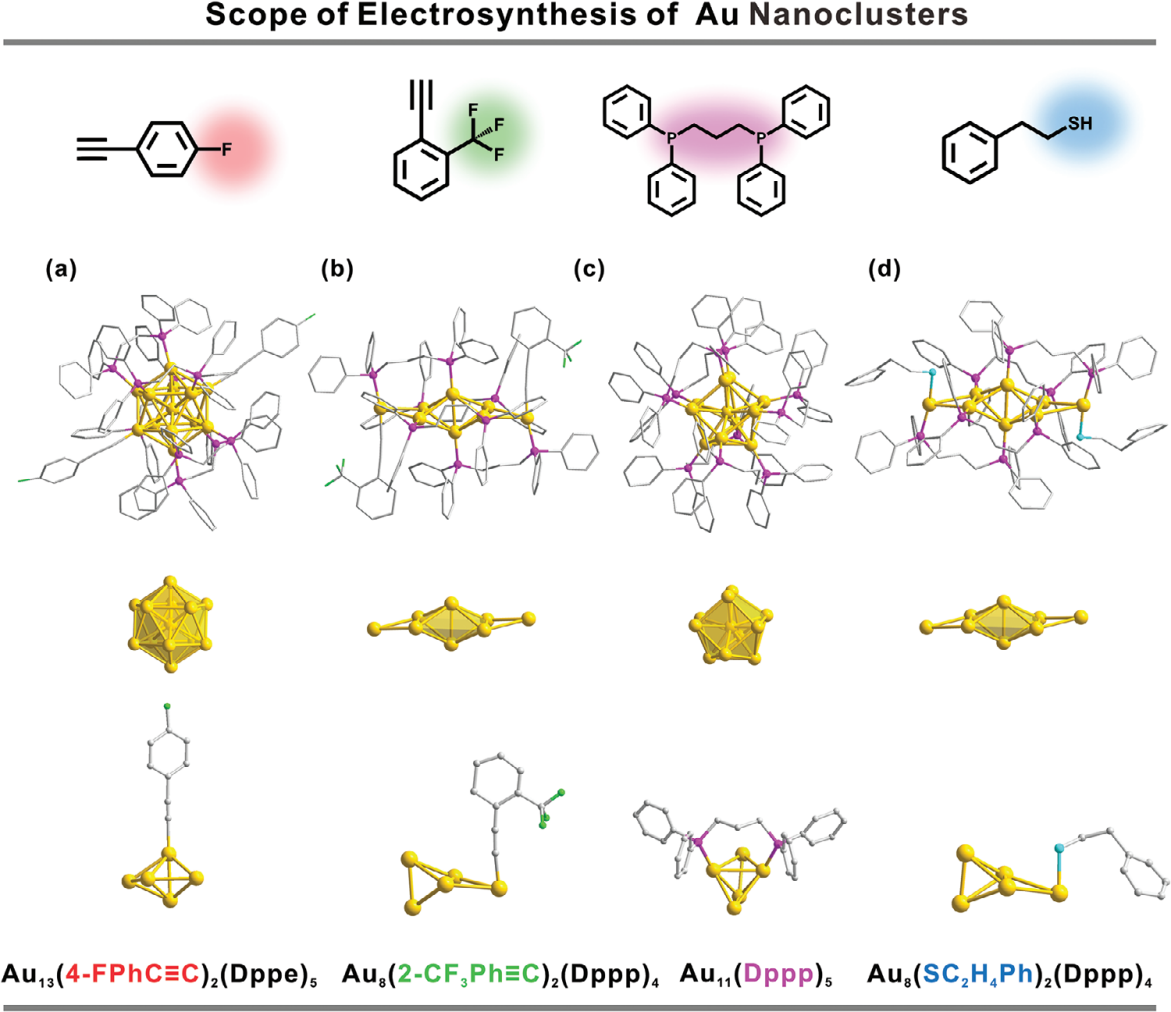
Scope of application. The structure anatomy of [Au_13_(4‐FPhC≡C)_2_(Dppe)_5_]Cl_3_ (**Au_13_‐2**) a). [Au_8_(2‐CF_3_PhC≡C)_2_(Dppp)_4_](PF_6_)_2_ (**Au_8_‐1**) b). [Au_11_(Dppp)_5_]Cl_3_ (**Au_11_**) (c) and[Au_8_(SC_2_H_4_Ph)_2_(Dppp)_4_]Cl_2_ (**Au_8_‐2**) d). Color code: yellow, Au; pink, P; gray, C; green, F; blue, S. All hydrogen atoms are omitted for clarity.

If electrolytic cell is to be qualified as a new platform for exploiting novel NCs with atomic precision, a good range of applicability is a critical criterion. For that, different ligands were screened (Figures S20 and S21, Supporting Information). Replacing TBA with 4‐fluorophenylacetylene while keeping the other conditions the same yielded a [Au_13_(4‐FPhC≡C)_2_(Dppe)_5_]Cl_3_ NC (**Au_13_‐2)** (**Figure** **6**a). Despite the difference in alkynyl ligand, its overall structure was similar with that of **Au_13_**. ESI mass analysis shows an intense peak at m/z 1596.76, which is assigned to the [Au_13_(4‐FPhC≡C)_2_(Dppe)_5_]^3+^ NC (Figure S22a, Supporting Information). The unity spacing of the isotopes indicates that the [Au_13_(4‐FPhC≡C)_2_(Dppe)_5_]^3+^ NC ion bears three charges. A combination of 1,3‐bis(diphenylphosphino)propane (Dppp) and 2‐(trifluoromethyl)phenylacetylene gave birth to a [Au_8_(2‐CF_3_PhC≡C)_2_(Dppp)_4_](PF_6_)_2_ NC (**Au_8_‐1**) (Figures 6b; and S23, Supporting Information). The core of **Au_8_** consists of an Au_6_ octahedral core and two extended gold atoms, each of which accommodates one phosphine and one alkynyl group (Figure 6b).^[^ ^69^ ^]^ By reducing the amount of alkynyl ligands, an all‐phosphine‐protected [Au_11_(Dppp)_5_]Cl_3_ NC (**Au_11_**) was synthesized (Figure 6c). **Au_11_** core can be described as a central gold atom surrounded by an icosahedron in which one triangle was replaced by a Au atom.^[^ ^70^ ^]^ Furthermore, thiolate has been more widely used than alkynyl for Au NCs. To test whether electrosynthesis is suitable for thiolate ligand, 2‐phenylethanethiol was used in place of alkynyl. A [Au_8_(SC_2_H_4_Ph)_2_(Dppp)_4_]Cl_2_ NC (**Au_8_‐2**) NC was acquired (Figure 6d). The difference between **Au_8_‐2** and **Au_8_‐1** is that the extended Au atom of Au_8_‐2 coordinates with a thiolate ligand other than alkynyl ligand.^[^ ^71^ ^]^ The mass peaks of [Au₈(2‐CF₃PhC≡C)₂(Dppp)₄]^2^⁺, [Au₁₁(Dppp)₅]^3^⁺, and [Au₈(SC₂H₄Ph)₂(Dppp)₄]^2^⁺ are observed at m/z 1781.72, 1409.48, and 1749.83, respectively (theoretical values: 1781.67, 1409.46, and 1749.72), as shown in Figures S22b and S22c (Supporting Information). They agree with the calculated isotopic patterns.

## 3 Conclusion

An electrochemical platform for synthesizing Au nanoclusters with atomic precision is established. The technical features, underlying mechanism, and scope of application are investigated. A tetranuclear intermediate Au(I) complex defines the redox behavior of the reaction solution. The critical role of base is to suppress the hydrogen evolution reaction at the cathode. By employing pulsed potential, the reaction rate and the yield of Au nanoclusters are significantly improved. The study aims to pushing the electrosynthesis to atom‐precision and inspiring future synthesis of various metal nanoclusters by this strategy.

## Conflict of Interest

The authors declare no conflict of interest.

## Supporting information



Supporting Information

## Data Availability

The data that support the findings of this study are available in the Supporting Information of this article.

## References

[1] M. C. Leech , K. Lam , Nat. Rev. Chem. 2022, 6, 275. 37117870 10.1038/s41570-022-00372-y

[2] C. Kingston , M. D. Palkowitz , Y. Takahira , J. C. Vantourout , B. K. Peters , Y. Kawamata , P. S. Baran , Acc. Chem. Res. 2020, 53, 72. 31823612 10.1021/acs.accounts.9b00539 PMC6996934

[3] M. Rafiee , D. J. Abrams , L. Cardinale , Z. Goss , A. Romero‐Arenas , S. S. Stahl , Chem. Soc. Rev. 2024, 5, 566. 10.1039/d2cs00706a PMC10842901 38050749

[4] L. F. T. Novaes , J. Liu , Y. Shen , L. Lu , J. M. Meinhardt , S. Lin , Chem. Soc. Rev. 2021, 50, 7941. 34060564 10.1039/d1cs00223f PMC8294342

[5] Y. Wang , S. Dana , H. Long , Y. Xu , Y. Li , N. Kaplaneris , L. Ackermann , Chem. Rev. 2023, 123, 11269. 37751573 10.1021/acs.chemrev.3c00158 PMC10571048

[6] Y. H. Budnikova , E. L. Dolengovski , M. V. Tarasov , T. V. Gryaznova , J. Solid State Electrochem. 2023, 28, 659.

[7] C. Zhu , N. W. J. Ang , T. H. Meyer , Y. Qiu , L. Ackermann , ACS Cent. Sci. 2021, 7, 415. 33791425 10.1021/acscentsci.0c01532 PMC8006177

[8] Y. Hioki , M. Costantini , J. Griffin , K. C. Harper , M. P. Merini , B. Nissl , Y. Kawamata , P. S. Baran , Science 2023, 380, 81. 37023204 10.1126/science.adf4762

[9] D. Lehnherr , L. Chen , Org. Process Res. Dev. 2024, 28, 338.

[10] T. H. Meyer , I. Choi , C. Tian , L. Ackermann , Chem 2020, 6, 2484.

[11] L. Zeng , Q. Yang , J. Wang , X. Wang , P. Wang , S. Wang , S. Lv , S. Muhammad , Y. Liu , H. Yi , A. Lei , Science 2024, 38, 216. 10.1126/science.ado0875 38991063

[12] Y. Yuan , J. Yang , A. Lei , Chem. Soc. Rev. 2021, 50, 10058. 34369504 10.1039/d1cs00150g

[13] M. Yan , Y. Kawamata , P. S. Baran , Chem. Rev. 2017, 117, 13230. 28991454 10.1021/acs.chemrev.7b00397 PMC5786875

[14] N. Wang , S. Ma , P. Zuo , J. Duan , B. Hou , Adv. Sci. 2021, 8, 2100076. 10.1002/advs.202100076 PMC8336511 34047062

[15] I. Saldan , O. Dobrovetska , L. Sus , O. Makota , O. Pereviznyk , O. Kuntyi , O. Reshetnyak , J. Solid State Electrochem. 2018, 22, 637.

[16] J. Hong , X. Su , J. Am. Chem. Soc. 2024, 146, 18586. 38949127 10.1021/jacs.4c04826

[17] N. Tian , Z.‐Y. Zhou , S.‐G. Sun , Y. Ding , Z. L. Wang , Science 2007, 316, 732. 17478717 10.1126/science.1140484

[18] L. Zhao , Y. Wang , S. Jin , N. An , M. Yan , X. Zhang , Z. Hong , S. Yang , Nat. Synth. 2024, 3, 867.

[19] M. W. Glasscott , A. D. Pendergast , S. Goines , A. R. Bishop , A. T. Hoang , C. Renault , J. E. Dick , Nat. Commun. 2019, 10, 2650. 31201304 10.1038/s41467-019-10303-z PMC6570760

[20] S. Butcha , S. Assavapanumat , S. Ittisanronnachai , V. Lapeyre , C. Wattanakit , A. Kuhn , Nat. Commun. 2021, 12, 1314. 33637758 10.1038/s41467-021-21603-8 PMC7910542

[21] D. Ramimoghadam , S. Bagheri , S. B. A. Hamid , J. Magn. Mater. 2014, 368, 207.

[22] N. An , T. Chen , J. Zhang , G. Wang , M. Yan , S. Yang , Small Methods 2024, 8, 2300910. 10.1002/smtd.202300910 38415973

[23] S. Cestellos‐Blanco , H. Zhang , J. M. Kim , Y. Shen , P. Yang , Nat. Catal. 2020, 3, 245.

[24] K. Rajeshwar , A. Vali , A. Rawat , N. Myung , Acc. Chem. Res. 2023, 56, 994. 37074812 10.1021/acs.accounts.2c00838

[25] N. K. Mahenderkar , Q. Chen , Y.‐C. Liu , A. R. Duchild , S. Hofheins , E. Chason , J. A. Switzer , Science 2017, 35, 1203. 10.1126/science.aam5830 28302857

[26] H. Masuda , K. Fukuda , Science 1995, 268, 1466. 17843666 10.1126/science.268.5216.1466

[27] H. Yao , J. Duan , D. Mo , H. Yusuf Günel , Y. Chen , J. Liu , T. Schäpers , J. Appl. Phys. 2011, 11, 094301.

[28] M. A. Saucedo‐Espinosa , M. Breitfeld , P. S. Dittrich , Angew. Chem., Int. Ed. 2023, 6, e202212459. 10.1002/anie.202212459 PMC10107445 36350110

[29] L. Zhang , R. Hao , D. Zhang , H. You , Y. Dai , W. Liu , J. Fang , Anal. Chem. 2020, 92, 9838. 32539342 10.1021/acs.analchem.0c01333

[30] M. M. Modena , B. Rühle , T. P. Burg , S. Wuttke , Adv. Mater. 2019, 31, 1901556. 10.1002/adma.201901556 31148285

[31] B. S. González , M. J. Rodríguez , C. Blanco , J. Rivas , M. A. López‐Quintela , J. M. Gaspar Martinho , Nano Lett. 2010, 10, 4217. 20836542 10.1021/nl1026716

[32] B. S. González , M. C. Blanco , M. A. López‐Quintela , Nanoscale 2012, 4, 7632. 23064311 10.1039/c2nr31994b

[33] J. Kong , W. Zhang , Y. Wu , M. Zhou , Aggregate 2022, 3, e207.

[34] J. Olesiak‐Banska , M. Waszkielewicz , P. Obstarczyk , M. Samoc , Chem. Soc. Rev. 2019, 48, 4087. 31292567 10.1039/c8cs00849c

[35] Z. Liu , L. Luo , R. Jin , Adv. Mater. 2024, 36, 2309073. 10.1002/adma.202309073 37922431

[36] X. Cai , G. Li , W. Hu , Y. Zhu , ACS Catal. 2022, 12, 10638.

[37] Z. Guan , J. Li , F. Hu , Q. Wang , Angew. Chem., Int. Ed. 2022, 61, e202209725. 10.1002/anie.202209725 36169269

[38] R. H. Adnan , J. M. L. Madridejos , A. S. Alotabi , G. F. Metha , G. G. Andersson , Adv. Sci. 2022, 9, 2105692. 10.1002/advs.202105692 PMC9130904 35332703

[39] F. Gao , Q. Yuan , P. Cai , L. Gao , L. Zhao , M. Liu , Y. Yao , Z. Chai , X. Gao , Adv. Sci. 2019, 6, 1801671. 10.1002/advs.201801671 PMC6446600 30989021

[40] H. Zhao , C. Zhang , B. Han , Z. Wang , Y. Liu , Q. Xue , C.‐H. Tung , D. Sun , Nat. Synth. 2024, 3, 517.

[41] X. Kang , H. Chong , M. Zhu , Nanoscale 2018, 10, 10758. 29873658 10.1039/c8nr02973c

[42] S. Kenzler , A. Schnepf , Chem. Sci. 2021, 1, 3116. 10.1039/d0sc05797e PMC8179421 34164079

[43] N. Xia , Z. Wu , Chem. Sci. 2021, 12, 2368. 10.1039/d0sc05363e PMC8179260 34164001

[44] C. Zeng , Y. Chen , K. Kirschbaum , K. Appavoo , M. Y. Sfeir , R. Jin , Sci. Adv. 2015, 1, e1500045. 26601152 10.1126/sciadv.1500045 PMC4643822

[45] B. Santiago‐Gonzalez , A. Monguzzi , J. M. Azpiroz , M. Prato , S. Erratico , M. Campione , R. Lorenzi , J. Pedrini , C. Santambrogio , Y. Torrente , F. De Angelis , F. Meinardi , S. Brovelli , Science 2016, 353, 571. 27493181 10.1126/science.aaf4924

[46] C. Sun , B. K. Teo , C. Deng , J. Lin , G.‐G. Luo , C.‐H. Tung , D. Sun , Coord. Chem. Rev. 2021, 427, 213576.

[47] B. Zhang , J. Chen , Y. Cao , O. J. H. Chai , J. Xie , Small 2021, 17, 2004381. 10.1002/smll.202004381 33511773

[48] L. Wang , J. Du , J. Wu , Z. A. Nan , S. Li , X. Tang , Z. Xie , Q. Xu , X. Gong , J. He , R. Chen , H. Shen , Adv. Sci. 2024, 12, 2410796. 10.1002/advs.202410796 PMC11714199 39498856

[49] M. Zhu , E. Lanni , N. Garg , M. E. Bier , R. Jin , J. Am. Chem. Soc. 2008, 130, 1138. 18183983 10.1021/ja0782448

[50] Y.‐X. Wang , J. Zhang , H.‐F. Su , X. Cui , C.‐Y. Wei , H. Li , X.‐M. Zhang , ACS Nano 2023, 17, 11607. 37288740 10.1021/acsnano.3c02005

[51] Z.‐M. Zhu , Y. Zhao , H. Zhao , C. Liu , Y. Zhang , W. Fei , H. Bi , M.‐B. Li , Nano Lett. 2023, 23, 7508. 37477210 10.1021/acs.nanolett.3c02026

[52] T. U. B. Rao , B. Nataraju , T. Pradeep , J. Am. Chem. Soc. 2010, 132, 16304. 21033703 10.1021/ja105495n

[53] T. Udayabhaskararao , T. Pradeep , J. Phys. Chem. Lett. 2013, 4, 1553. 26282314 10.1021/jz400332g

[54] H. Xu , K. S. Suslick , ACS Nano 2010, 4, 3209. 20507161 10.1021/nn100987k

[55] I. Chakraborty , T. Udayabhaskararao , G. K. Deepesh , T. Pradeep , J. Mater. Chem. B 2013, 1, 4059. 32260958 10.1039/c3tb20603c

[56] S. Antonello , T. Dainese , F. Pan , K. Rissanen , F. Maran , J. Am. Chem. Soc. 2017, 139, 4168. 28281762 10.1021/jacs.7b00568

[57] M. Sugiuchi , Y. Shichibu , T. Nakanishi , Y. Hasegawa , K. Konishi , Chem. Commun. 2015, 51, 13519. 10.1039/c5cc04312c 26215256

[58] K. Kwak , V. D. Thanthirige , K. Pyo , D. Lee , G. Ramakrishna , J. Phys. Chem. Lett. 2017, 8, 4898. 28933858 10.1021/acs.jpclett.7b01892

[59] M. Kim , Q. Tang , A. V. Narendra Kumar , K. Kwak , W. Choi , D. Jiang , D. Lee , J. Phys. Chem. Lett. 2018, 9, 982. 29420895 10.1021/acs.jpclett.7b03261

[60] K. Kwak , D. Lee , Acc. Chem. Res. 2019, 52, 12. 30500153 10.1021/acs.accounts.8b00379

[61] U. Koelle , A. Laguna , Inorg. Chim. Acta 1999, 290, 44.

[62] M. B. Hariri , Anal. Methods 2021, 13, 2688. 34036981 10.1039/d1ay00361e

[63] S. Eggleston , F. Chodosh , R. Girard , D. T. Hill , Inorg. Chim. Acta 1985, 108, 221.

[64] M. Rezaei , S. H. Tabaian , D. F. Haghshenas , J. Electroanal. Chem. 2012, 687, 95.

[65] C. Su , M. An , P. Yang , H. Gu , X. Guo , Appl. Surf. Sci. 2010, 256, 4888.

[66] X. Li , H. Su , R. Zhou , S. Feng , Y. Tan , X. Wang , J. Jia , M. Kurmoo , D. Sun , L. Zheng , Chem.–Eur. J. 2016, 22, 3019. 26807553 10.1002/chem.201504799

[67] T. Liu , J. Wang , X. Yang , M. Gong , J. Energy Chem. 2021, 59, 69.

[68] G. González‐Rubio , A. Guerrero‐Martínez , L. M. Liz‐Marzán , Acc. Chem. Res. 2016, 49, 678. 27035211 10.1021/acs.accounts.6b00041 PMC4838951

[69] N. Kobayashi , Y. Kamei , Y. Shichibu , K. Konishi , J. Am. Chem. Soc. 2013, 135, 16078. 24127776 10.1021/ja4099092

[70] J. M. M. Smits , J. J. Bour , F. A. Vollenbroek , P. T. Beurskens , J. Crystallogr. Spectrosc. Res. 1983, 13, 355.

[71] M. Iwasaki , N. Kobayashi , Y. Shichibu , K. Konishi , Phys. Chem. Chem. Phys. 2016, 18, 19433. 27378218 10.1039/c6cp03129c

