# Supplementary material for: Electrosynthesis of Atomically Precise Au Nanoclusters
Source: Adv Sci (Weinh). 2025 Mar 13;12(17):2414057. doi: 10.1002/advs.202414057 (PMC12061246; doi:10.1002/advs.202414057)
Supplement: Supplementary file 1 — Supporting Information [file ADVS-12-2414057-s001.docx]

**Supporting Information**

**Electrosynthesis of Atomically Precise Au Nanoclusters**

Jing Dong, Yawei Li, Yu Ding, Hai-Feng Su, Xiaoqin Cui, Yu-Xin Wang, Huan Li^*^

**Table of Contents:**

**Part I. Materials and Methods**

- 1. Physical measurements
  2. Synthesis methods
  3. X-ray Crystallography

**Part Ⅱ. Supporting figures**

**Part Ⅲ. Crystallographic Supplementary Information**

**Part Ⅳ. References**

**Part I. Materials and Methods**

**1.1 Physical measurements and materials**

Fourier transform infrared (FT-IR) spectra were collected on Nicolet iS5 with samples prepared as KBr pellets. UV-vis absorption spectra were recorded on TU-1950 UV-vis spectrophotometer with samples being dispersed in CH_2_Cl_2_. ^1^H-NMR was acquired on a Bruker Avance III spectrometer (600MHz) using CD_3_OD as solvent. The ESI-MS analysis was performed on an ESI-TOF mass spectrometer (6224, Agilent). The gas chromatography (GC) was performed by an Agilent 7890B. Electrochemical measurements were performed with a CHI660E electrochemical workstation. The working electrode was glassy carbon (GC) electrode, Pt or Ni plate. Pt wire or graphite electrode was used as the counter electrode, and saturated calomel electrode (SCE) or Ag/AgCl electrode as reference electrode. Powder X-ray diffraction (PXRD) analysis was performed on a Rigaku Ultima IV diffractometer configured with a Cu-Kα radiation source (λ = 1.5418 Å) and graphite monochromator at 40 kV voltage and 40 mA current.

**1.2 Synthesis methods**

**Synthesis of** **[Au_13_(4-*^t^*BuPhC≡C)_2_(Dppe)_5_]Cl_3_**

The electrosynthesis of [Au_13_(4-*^t^*BuPhC≡C)_2_(Dppe)_5_]Cl_3_ (**Au_13_**) was conducted in a single electrolytic cell using a graphite electrode (ϕ 0.6 × 0.75 cm) as anode and Ni plate (1 × 1 × 0.02 cm) as cathode. Before synthesis, the graphite electrode was polished with sandpaper and ultrasonically rinsed with ethanol for 5 minutes. The Ni electrode was polished with sandpaper and followed by cleaning with ethanol. Tetrabutylammonium hexafluorophosphate (0.1 M Bu_4_NPF_6_) was used as supporting electrolyte.

HAuCl_4_ (0.05 mmol) was dissolved in 2 mL ethanol, into which Me_2_S (15 μL) was charged. Next, a CH_2_Cl_2_ solution (2 mL) of 4-***^t^***BuPhC≡CH (0.05 mmol, 9 μL) and Dppe (1,2-bis- (diphenylphosphino)ethane, 0.025 mmol, 10 mg) was added dropwise. Et_3_N (30 μL) and electrolyte Bu_4_NPF_6_ (190 mg, 0.5 mmol) were then fed. Under a constant potential of −3 V, the color of the solution gradually changed from colorless to dark brown in 24 hours. The mixture was evaporated to dryness, yielding a dark brown solid, which was dissolved in CH_2_Cl_2_ (2 mL). The solution was centrifuged for 10 min at 12000 r/min. The supernatant solution was subject to the diffusion with *n*-hexane and diethyl ether (volume ratio, 1:1) to afford the red block-shaped crystals after one week.

**Synthesis of** **[Au_13_(4-FPhC≡C)_2_(Dppe)_5_]Cl_3_**

The synthesis and crystal growth conditions of [Au_13_(4-FPhC≡C)_2_(Dppe)_5_]Cl_3_ (**Au_13_-2**) were the same as [Au_13_(4-*^t^*BuPhC≡C)_2_(Dppe)_5_]Cl_3_ (**Au_13_**) (isolated yield 27%), except that 4-FPhC≡CH was used instead of 4-*^t^*BuPhC≡CH.

**Synthesis of** **[Au_8_(2-CF_3_PhC≡C)_2_(Dppp)_4_](PF_6_)_2_**

The synthesis and crystal growth conditions of [Au_8_(2-CF_3_PhC≡C)_2_(Dppp)_5_]Cl_3_ (**Au_8_-1**) (isolated yield 20%) were the same as [Au_13_(4-*^t^*BuPhC≡C)_2_(Dppe)_5_]Cl_3_ (**Au_13_**), except that 4-*^t^*BuPhC≡CH was used instead of 2-CF_3_PhC≡CH, Dppe (1,2-bis- (diphenylphosphino)ethane) was used instead of Dppp (1,3-bis- (diphenylphosphino)propane).

**Synthesis of** **[Au_11_(Dppp)_5_]Cl_3_**

The synthesis and crystal growth conditions of [Au_11_(Dppp)_5_]Cl_3_ **(Au_11_)** (isolated yield 23%) were the same as [Au_8_(2-CF_3_PhC≡C)_2_(Dppp)_5_]Cl_3_ (**Au_8_-1**), except that 2-CF_3_PhC≡CH (0.05 mmol, 9 μL) was used instead of 3-CF_3_PhC≡CH (0.025 mmol, 4 μL).

**Synthesis of** **[Au_8_(SC_2_H_4_Ph)_2_(Dppp)_4_]Cl_2_**

The synthesis conditions of [Au_8_(SC_2_H_4_Ph)_2_(Dppp)_4_]Cl_2_ (**Au_8_-2**) were similar to [Au_8_(2-CF_3_PhC≡C)_2_(Dppp)_5_]Cl_3_ (**Au_8_-1**), except that 2-CF_3_PhC≡CH was used instead of SC_2_H_4_Ph. **Au_8_-2** was synthesized under a constant potential of −5.0 V. The reaction mixture was stirred at room temperature for 24 hours. The mixture was evaporated to dryness, yielding a dark brown solid, which was then dissolved in CH_2_Cl_2_ (2 mL). The solution was centrifuged for 10 min at 12000 r/min. The supernatant solution was subject to the diffusion with *n*-hexane to afford pink flake crystals after one week (isolated yield 22%).

**1.3 X-ray Crystallography**

Single crystal X-ray diffraction data was collected on an Agilent Technologies SuperNova Single Crystal diffractometer equipped with a CCD area detector using Cu Kα radiation (λ = 1.54178 Å). Absorption corrections were applied by using the program CrysAlis^[1]^ (multi-scan). The structure was solved and refined using Full-matrix least-squares based on F^2^ with program^[2]^ SHELXS-97 and SHELXL-97 within OLEX2^[3]^. Non-hydrogen atoms were refined anisotropically. All remaining hydrogen atoms were added at calculated positions and refined by use of riding models with isotropic displacement parameters based on those of the parent atoms. Appropriate restraints or constraints were applied to the geometry and the atomic displacement parameters of the atoms in six crystals. Six structures were examined using the Addsym subroutine of PLATON to ensure that no additional symmetry could be applied to the models. Pertinent crystallographic data collection and refinement parameters were collated in Supplementary Table 1 to Table 6.

**Part Ⅱ. Supporting figures**

**
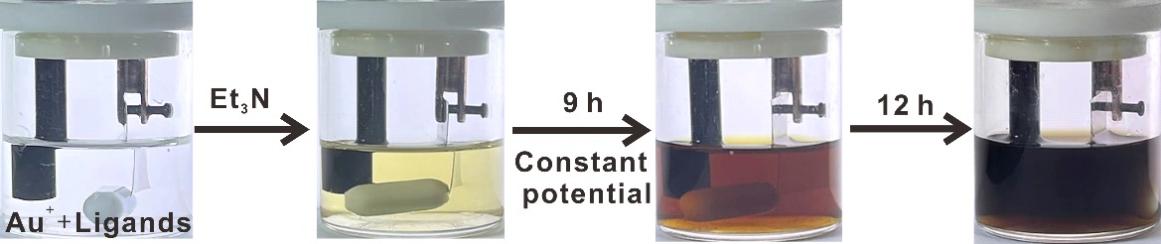
**

**Figure S1** Color change of the solution during the electrosynthesis of [Au_13_(4-*^t^*BuPhC≡C)_2_(Dppe)_5_]Cl_3_ nanocluster (**Au_13_**). Reaction conditions: HAuCl_4_ (0.05 mmol), 4-*tert*-butylphenylacetylene (0.05 mmol), and 1,2-bis-(diphenylphosphino)ethane (0.025 mmol), Et_3_N (30 μL); 0.1 M Bu_4_NPF_6_; graphite electrode and Ni plate were used as anode and cathode, respectively.


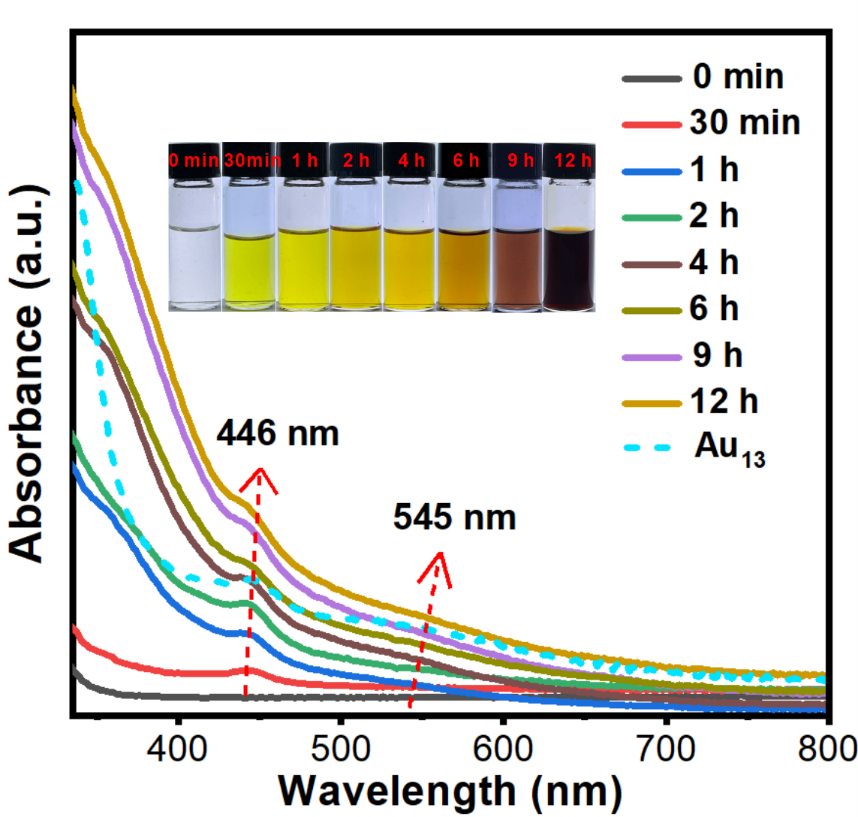


**Figure S2** UV-vis absorption spectra tracing of the reaction solution under a constant potential of −3 V. (0 min: UV–vis absorption spectrum of the solution before electrosynthesis)

**Figure S3** Stability test under working conditions. Reaction conditions: as-prepared **Au_13_** reaction solution; graphite electrode and Ni plate were used as anode and cathode, respectively.


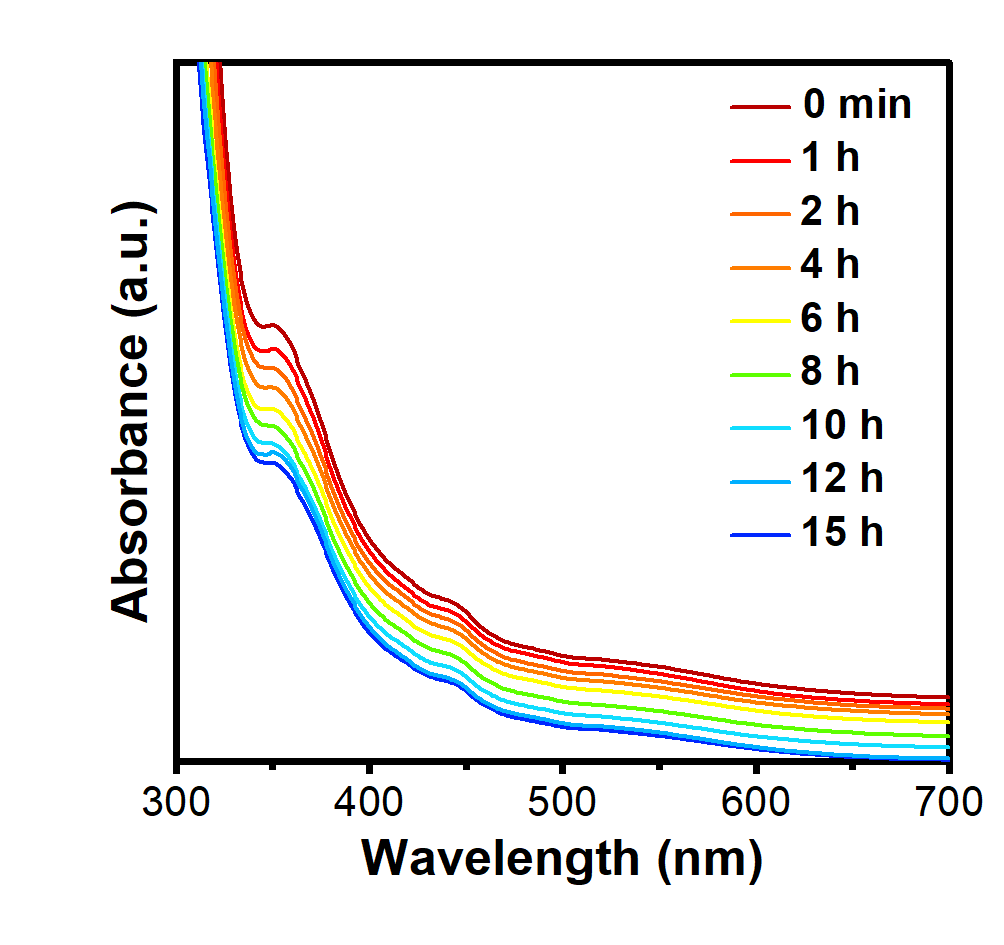


**Figure S4** Stability test. UV-vis absorption spectra tracing of the reaction solution. (0 min: UV-vis absorption spectrum of the reaction solution after electrosynthesis and aging)


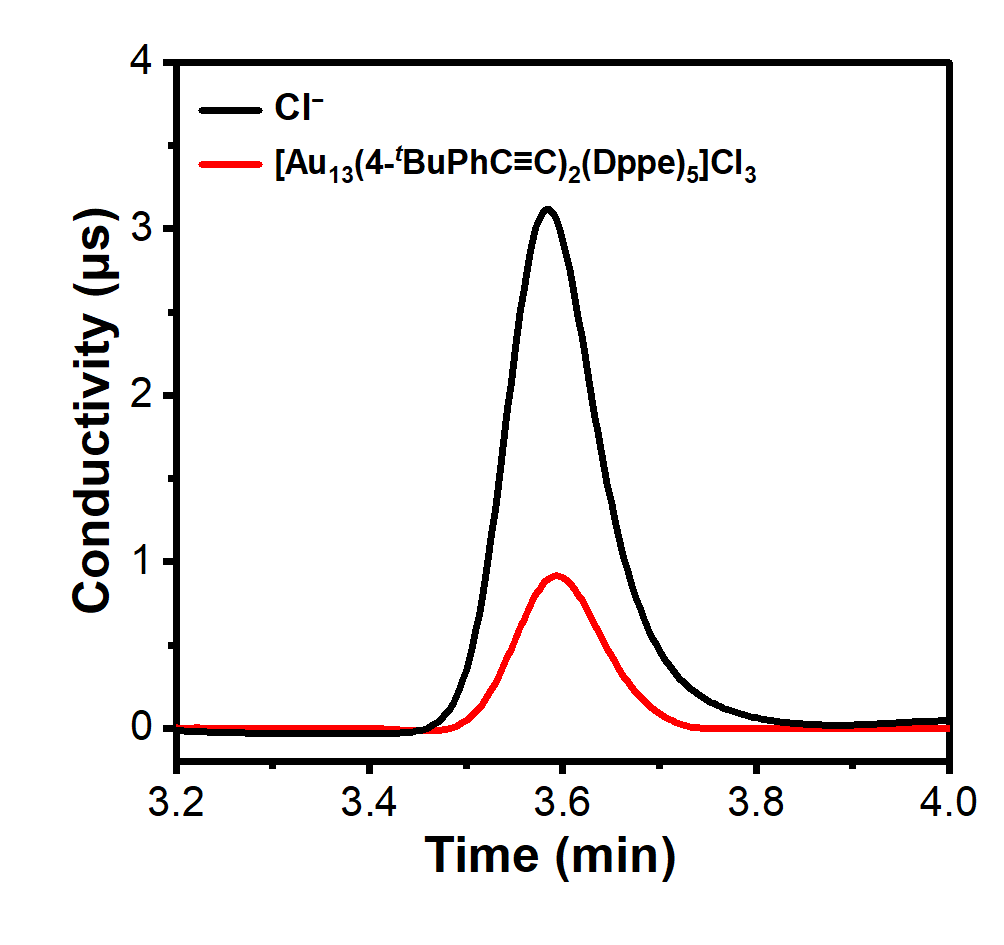


**Figure S5** The extraction of ion chromatogram of [Au_13_(4-*^t^*BuPhC≡C)_2_(Dppe)_5_]Cl_3_ (**Au_13_**) (red) with Cl⁻ (black) as a reference.


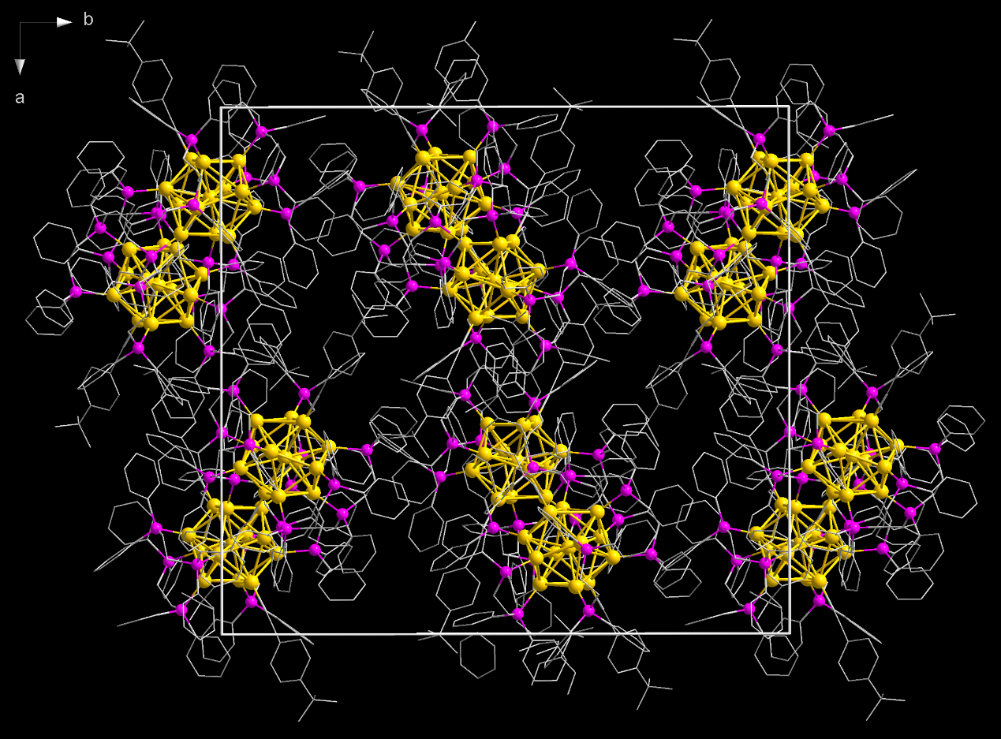


**Figure S6** Packing of **Au_13_** clusters in a unit cell, viewed along c-axis. Color legend: Au, yellow; P, purple; C, gray. Hydrogen atoms are omitted for clarity.


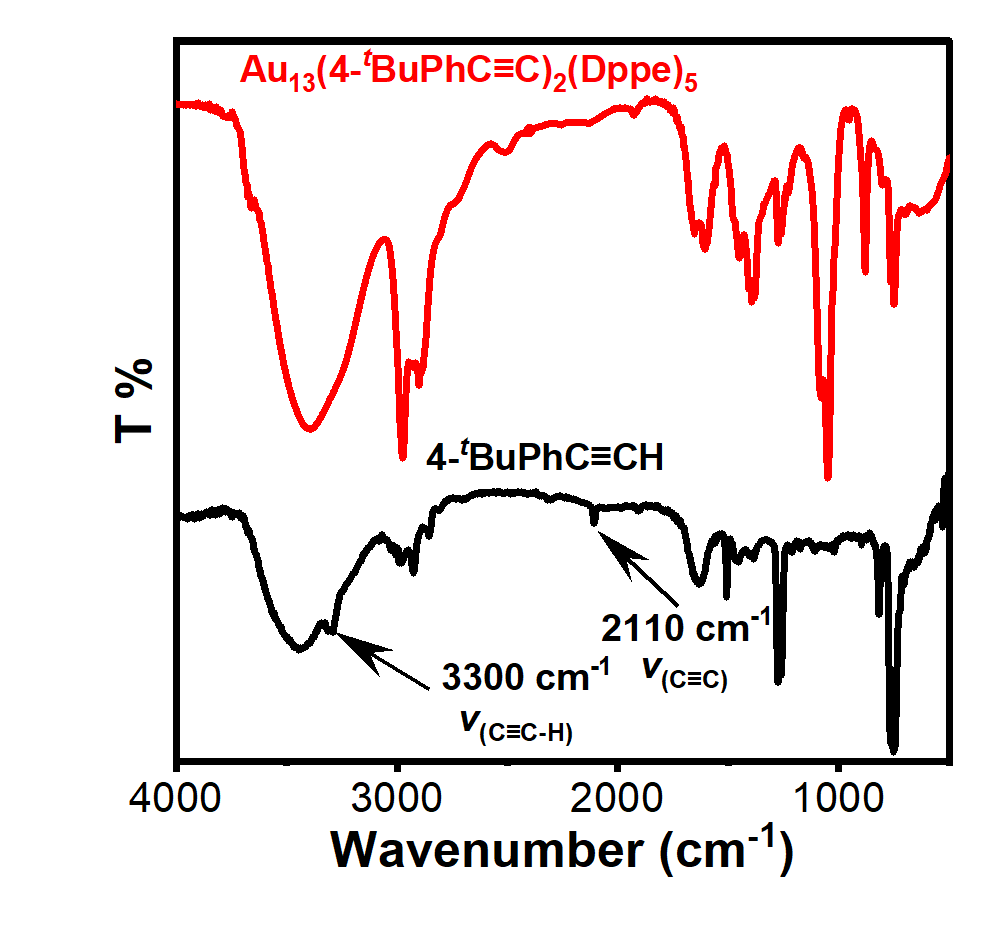


**Figure S7** Infrared spectra of **Au_13_** single crystal (red line) and 4-*tert*-butylphenylacetylene (black line). The absorption at 2110 cm^-1^ and 3300cm^-1^ was ascribed to C≡C and C≡C−H stretching modes, respectively.


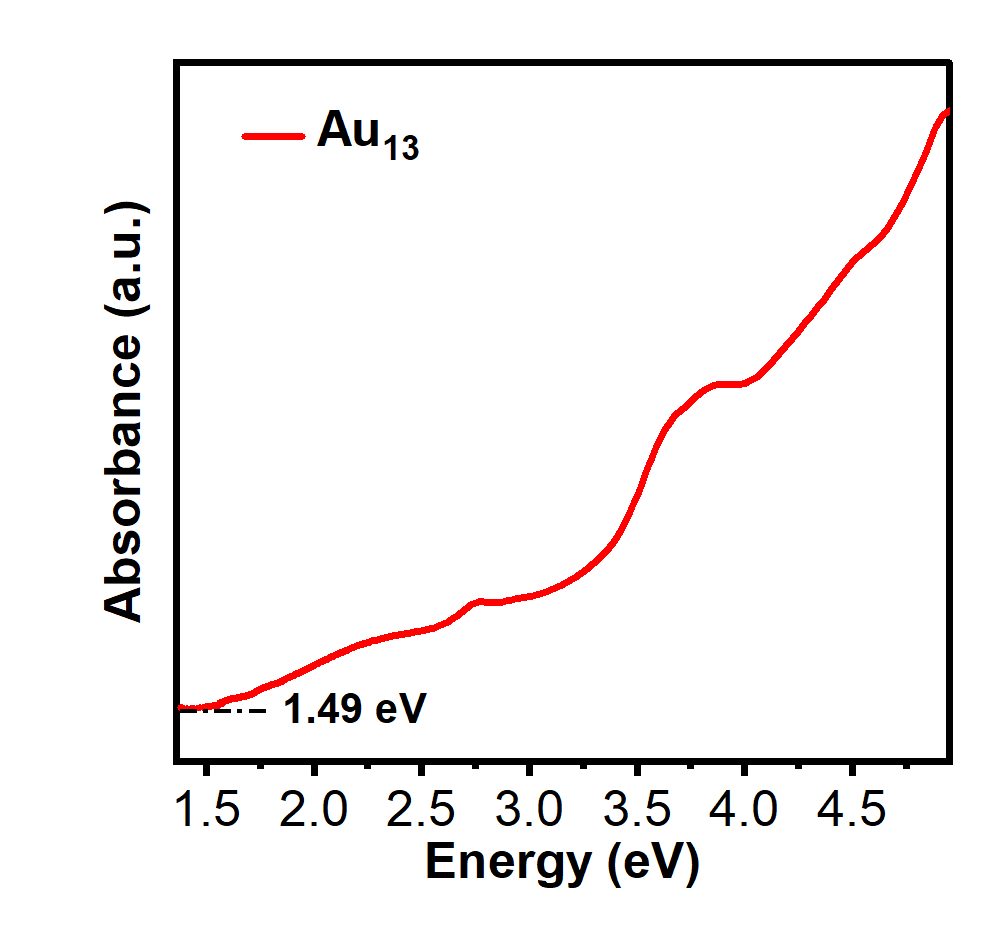


**Figure S8** UV−vis absorption spectrum of **Au_13_** at energy-scale. The optical band gap was calculated to be 1.49 eV.


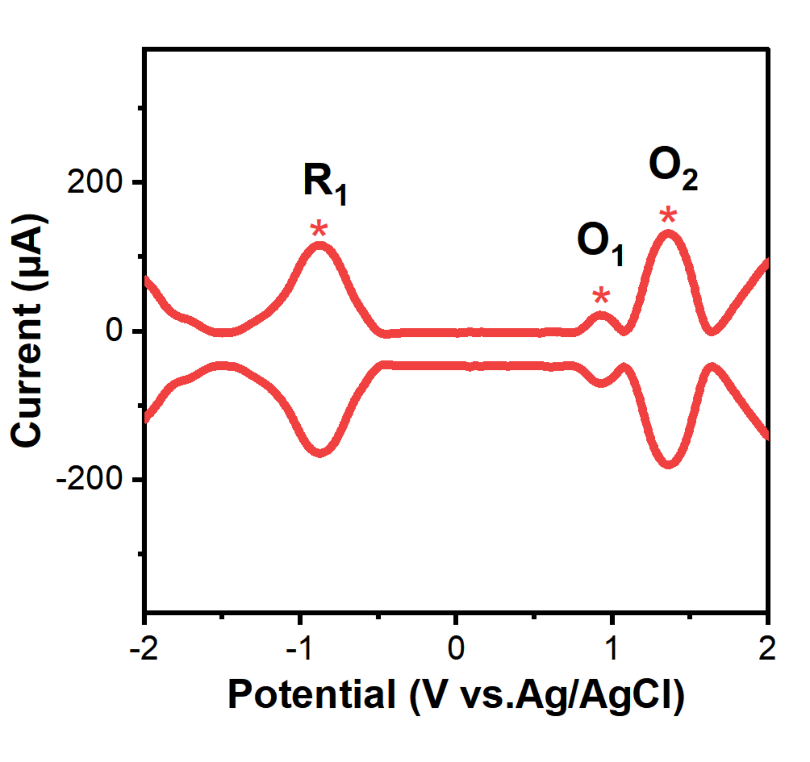


**Figure S9** Square wave voltammetry (SWV) of **Au_13_** in CH_2_Cl_2_ containing 0.1 M Bu_4_NPF_6_. Working electrode: Pt electrode; counter electrode: Pt wire; reference electrode: Ag/AgCl electrode. Two oxidation peaks at +0.94 V (O_1_) and +1.37 V (O_2_), and a reduction peak at −0.87 V (R_1_), with corresponding to an electrochemical gap of 1.81 V. The HOMO–LUMO gap was 1.38 eV.


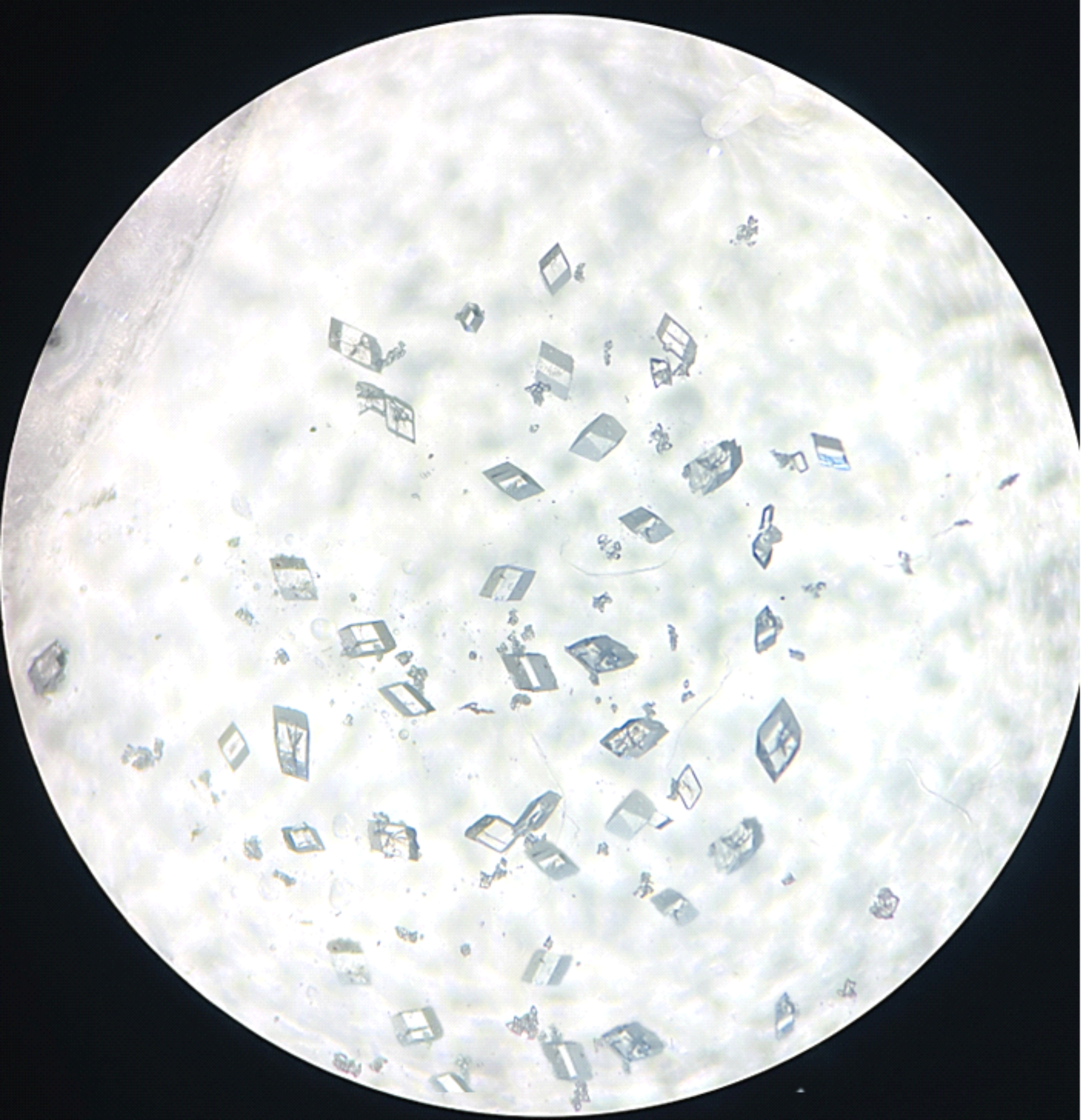


**Figure S****10** Single crystal photograph of the complex Au_4_(Dppe)_2_Cl_4_.


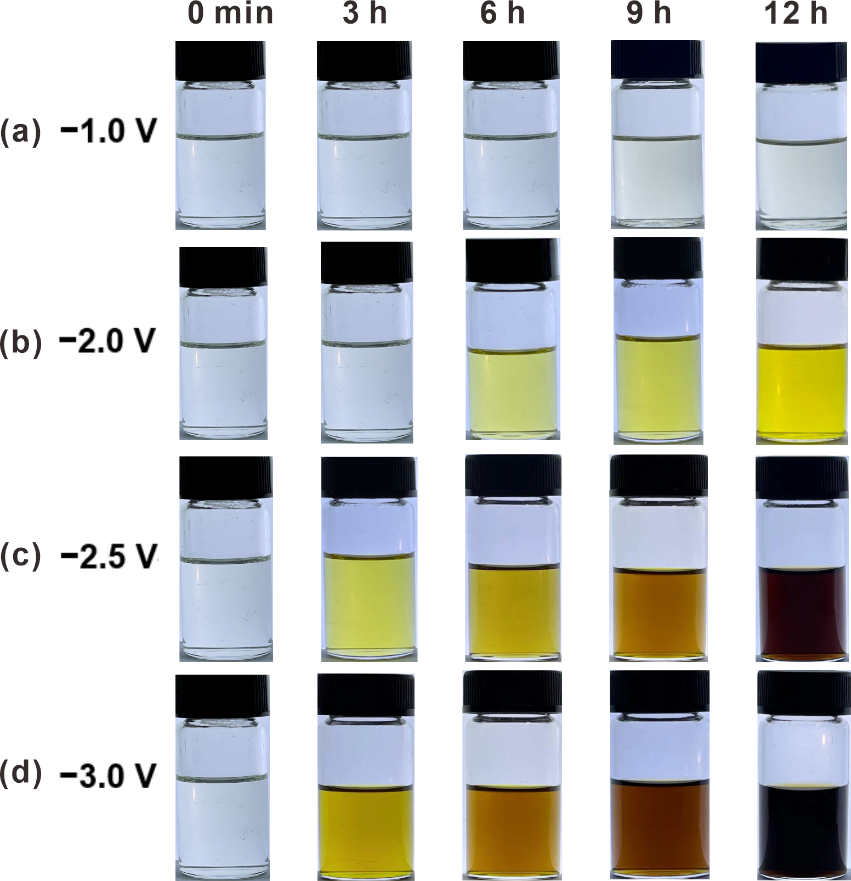


**Figure S11** Color changes of the solutions under different constant potential. Reaction conditions: HAuCl_4_ (0.05 mmol), 4-*tert*-butylphenylacetylene (0.05 mmol), and 1,2-bis-(diphenylphosphino)ethane (0.025 mmol), Et_3_N (30 μL); 0.1 M Bu_4_NPF_6_; graphite electrode and Ni plate were used as anode and cathode, respectively.


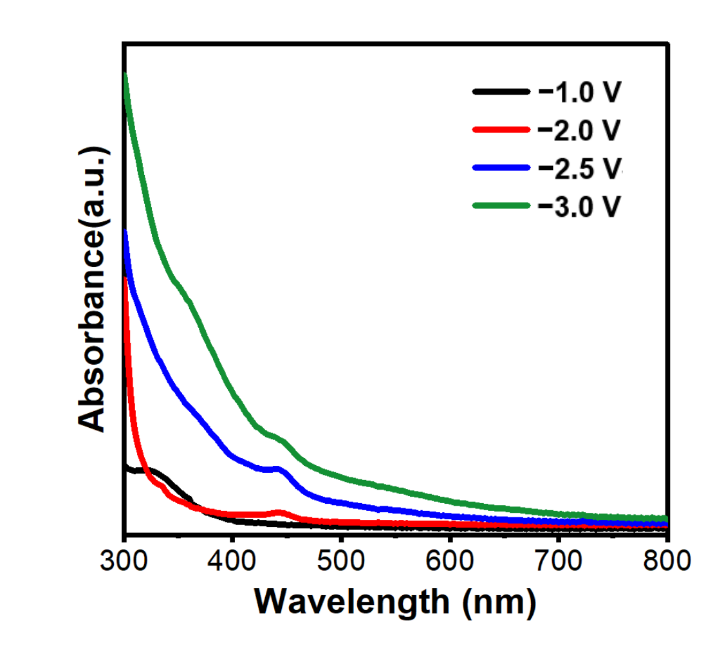


**Figure S12** UV-vis absorption spectra of **Au_13_** in CH_2_Cl_2_ for 12 hours under different potentials. Reaction conditions: HAuCl_4_ (0.05 mmol), 4-*tert*-butylphenylacetylene (0.05 mmol), and 1,2-bis-(diphenylphosphino)ethane (0.025 mmol), Et_3_N (30 μL); 0.1 M Bu_4_NPF_6_; graphite electrode and Ni plate were used as anode and cathode, respectively.


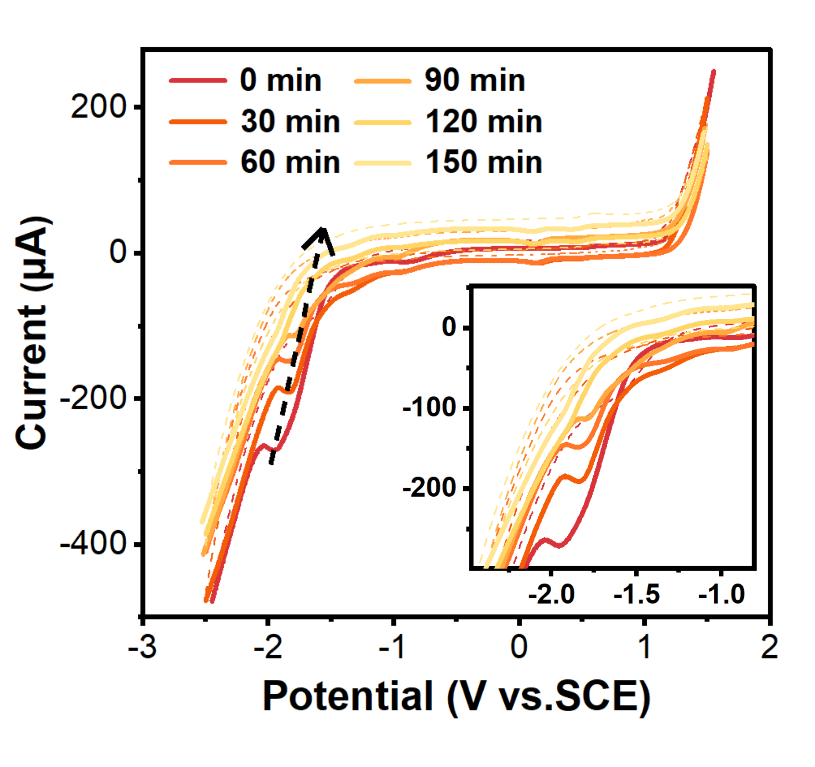


**Figure S13** Cyclic voltammetry (CV) tracking of the reaction solution during the electrosynthesis of **Au_13_** under a constant potential of −3 V. CV test conditions: working electrode: glassy carbon electrode, counter electrode: Pt wire, reference electrode: saturated calomel electrode; 0.1 M Bu_4_NPF_6_; Scan rate = 100 mV/s.


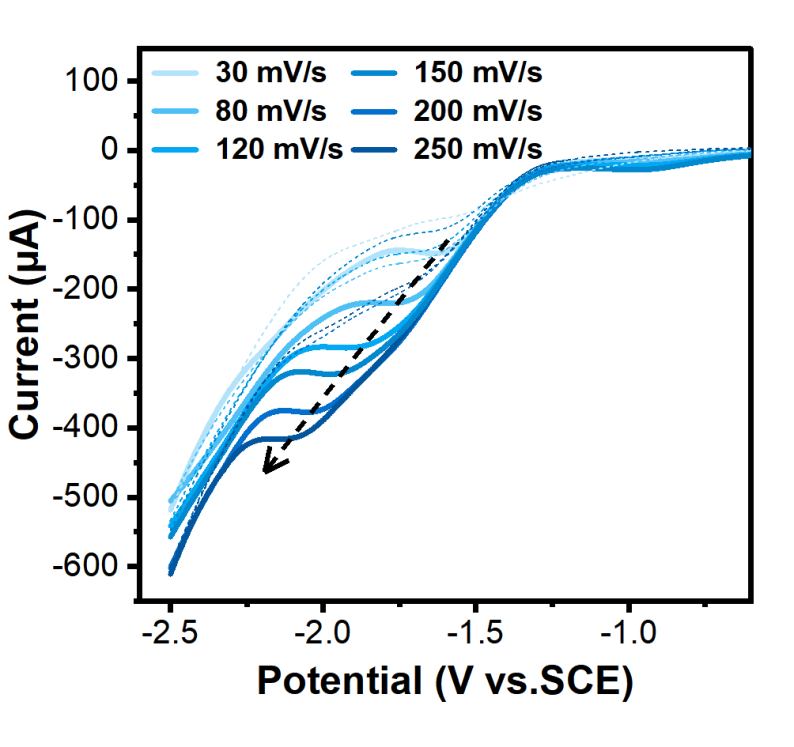


**Figure S14** CV of reaction solution at different scan rates. CV test conditions: working electrode: glassy carbon electrode, counter electrode: Pt wire, reference electrode: saturated calomel electrode; 0.1 M Bu_4_NPF_6._

_
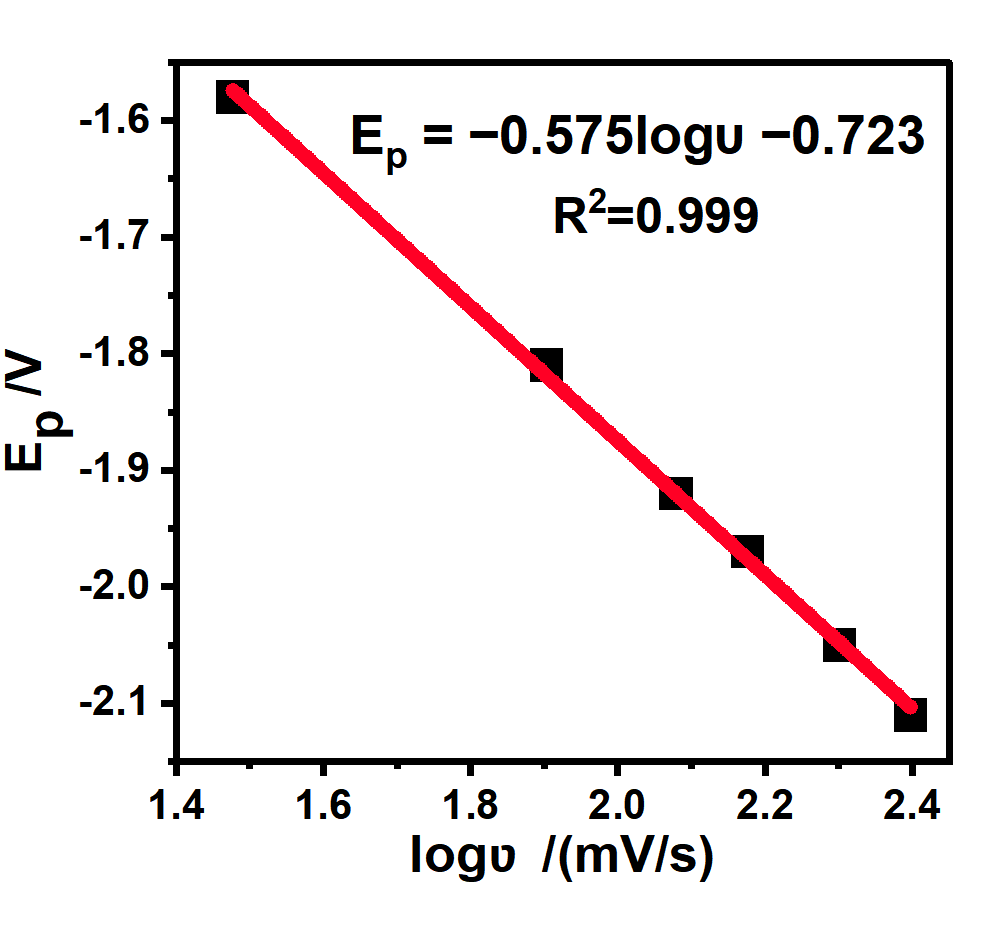
_

**Figure S15** The plot of the peak potential (E_p_) vs. log*υ* extracted from Figure S12. The reaction conditions were consistent with those in Figure S14.


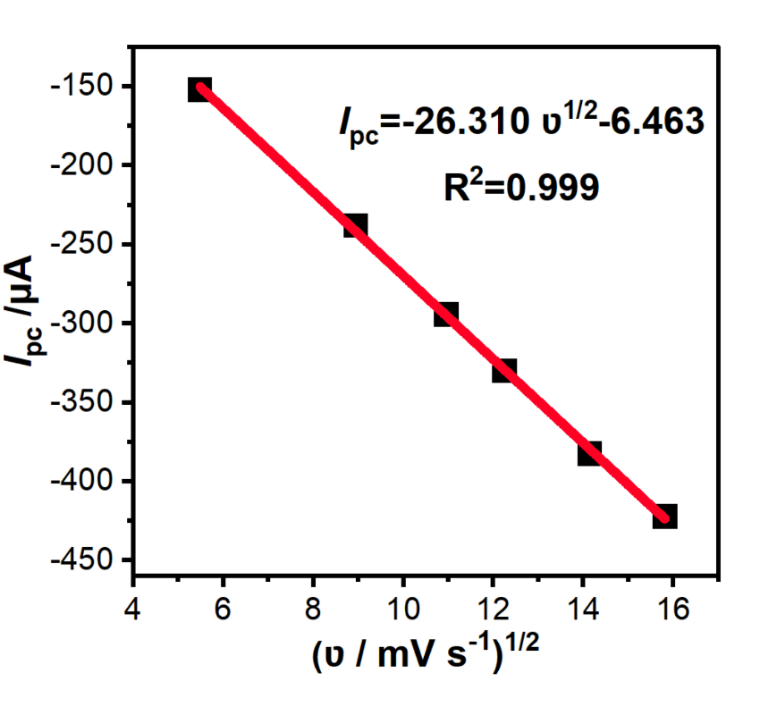


**Figure S16** The fitting results between the peak current I_p_ and the square root of the scanning rate *υ*^1/2^. The data was abstracted from Figure S14. The reaction conditions were consistent with those in Figure S14.


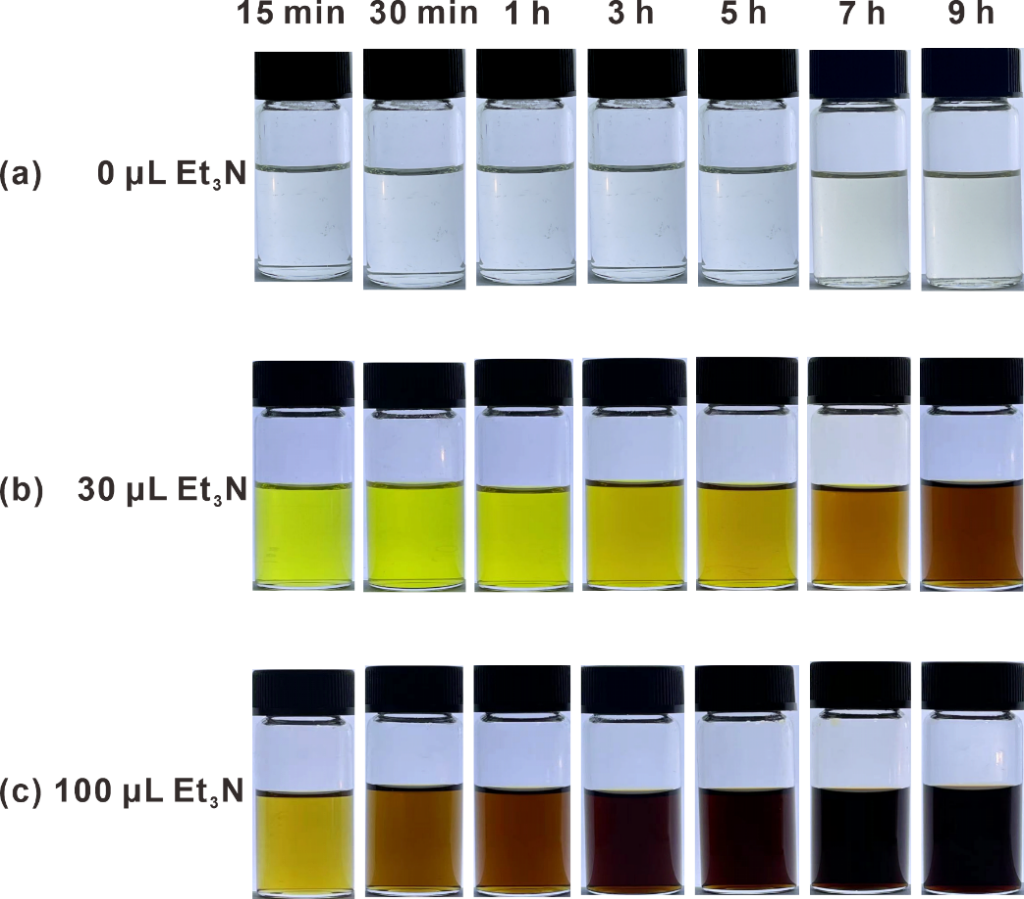


**Figure S17** Color changes of the solutions in the presence of different amounts of Et_3_N (a) 0 μL (b) 30 μL (c) 100 μL. Reaction conditions: Constant potential of −3 V; HAuCl_4_ (0.05 mmol), 4-*tert*-butylphenylacetylene (0.05 mmol), and 1,2-bis-(diphenylphosphino)ethane (0.025 mmol), 0.1 M Bu_4_NPF_6_; graphite electrode and Ni plate were used as anode and cathode, respectively.


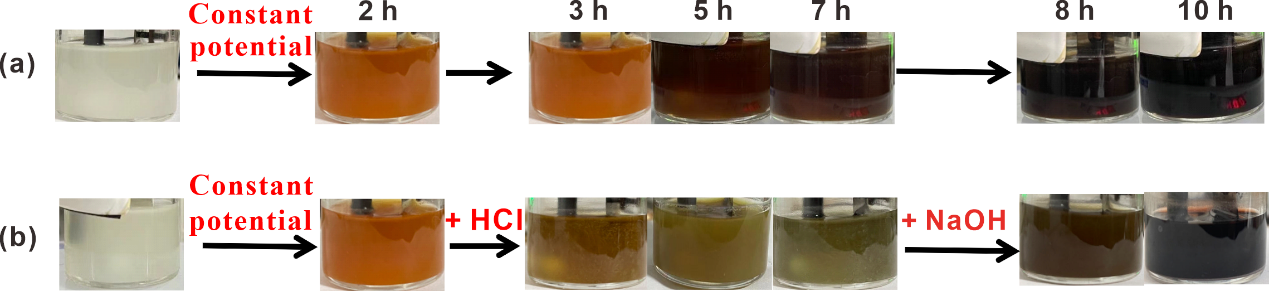


**Figure S1****8** (a) was the color change of the solution under a constant potential of −3 V. (b) After 2 hours of reaction, 100 μL 10 M HCl was charged into the vessel. In the following 5 hours, the solution color did not get darker. After the 7th hour of reaction, 1.0 mmol NaOH was introduced. The formation of Au nanocluster continued. Reaction conditions: HAuCl_4_ (0.05 mmol), 4-*tert*-butylphenylacetylene (0.05 mmol), and 1,2-bis-(diphenylphosphino)ethane (0.025 mmol), NaOH (0.5 mmol); 0.1 M Bu_4_NPF_6_; graphite electrode and Ni plate were used as anode and cathode, respectively.


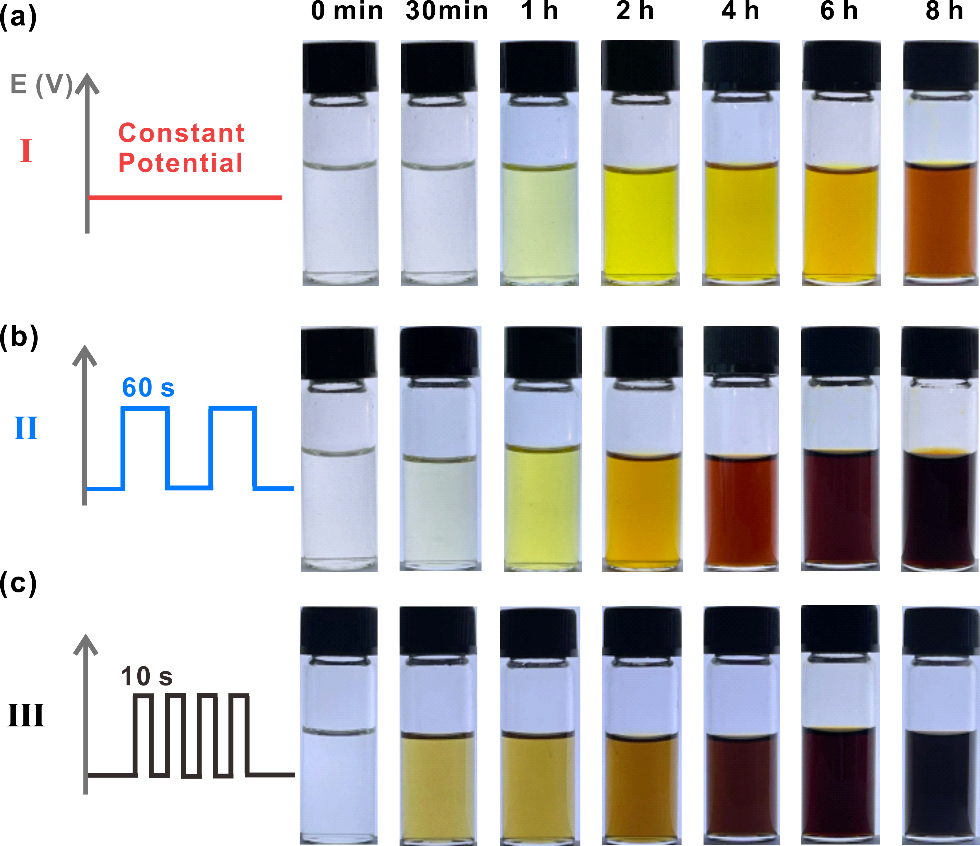


**Figure S19** Color changes of the solutions under (a) constant potential, (b) pulsed potential (pulse width 60 s), and (c) pulsed potential (pulse width 10 s). Reaction conditions: HAuCl_4_ (0.05 mmol), 4-*tert*-butylphenylacetylene (0.05 mmol), and 1,2-bis-(diphenylphosphino)ethane (0.025 mmol), Et_3_N (30 μL); 0.1 M Bu_4_NPF_6_; graphite electrode and Ni plate were used as counter electrode and working electrode, respectively.


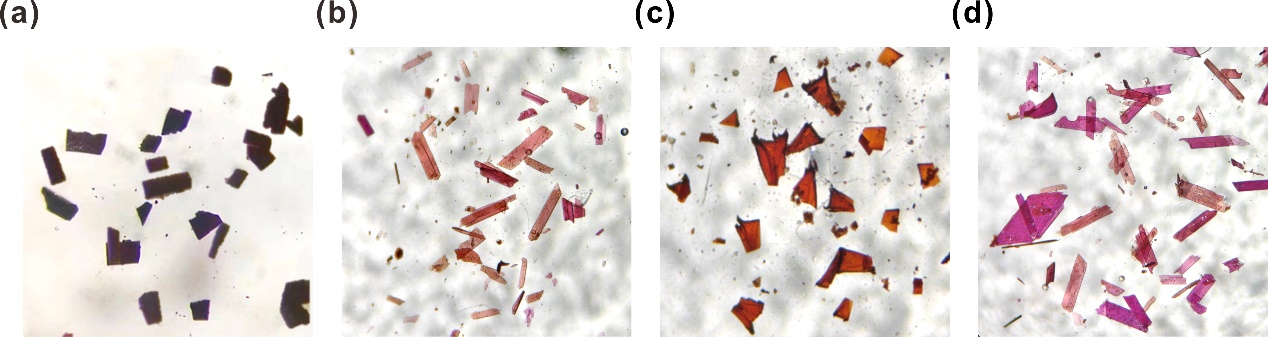
**Figure S****20** Photographs of single crystals of (a) **Au_13_-2** (b) **Au_8_-1** (c) **Au_11_** (d) **Au_8_-2**.

**
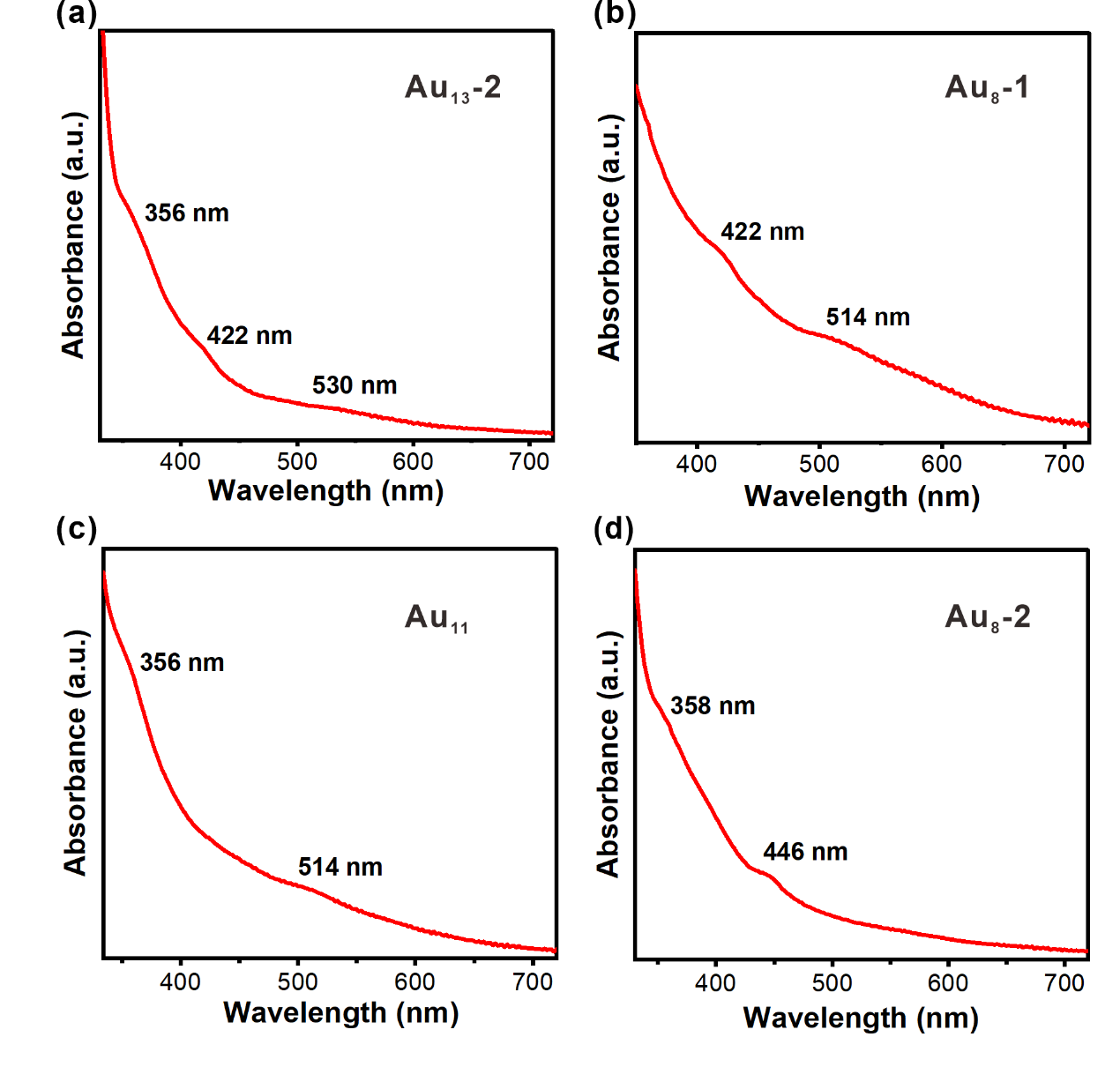
Figure S21** UV-vis absorption spectra of (a) **Au_13_-2** (b) **Au_8_-1** (c) **Au_11_** and (d) **Au_8_-2**.


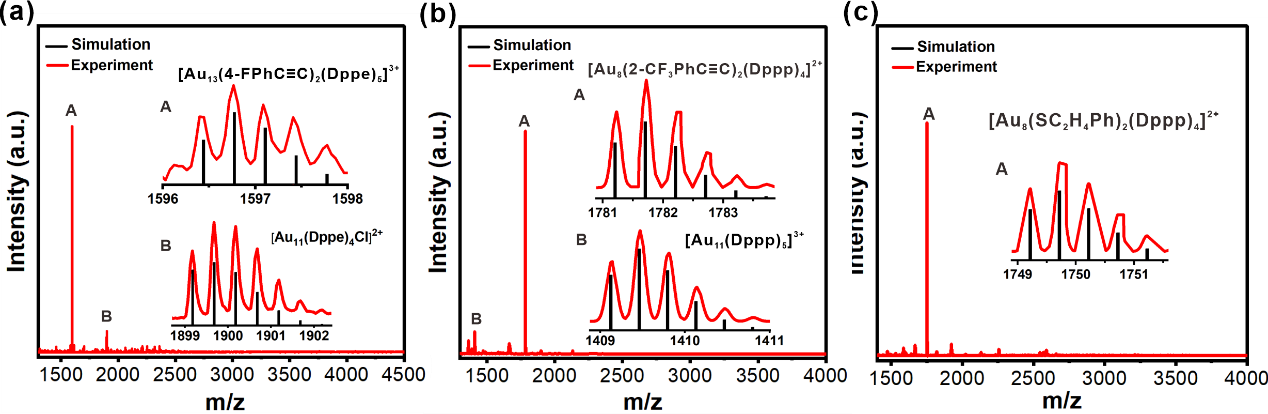


**Figure S22** Mass spectra of (a) [Au_13_(4-FPhC≡C)_2_(Dppe)_5_]Cl_3_ (**Au_13_-2**), (b) [Au_8_(2-CF_3_PhC≡C)_2_(Dppp)_4_](PF_6_)_2_ (**Au_8_-1**), and (c) [Au_8_(SC_2_H_4_Ph)_2_(Dppp)_4_]Cl_2_ (**Au_8_-2**). Inset: experimental (red trace) and simulated (black trace) isotopic patterns of the molecular ion peaks.


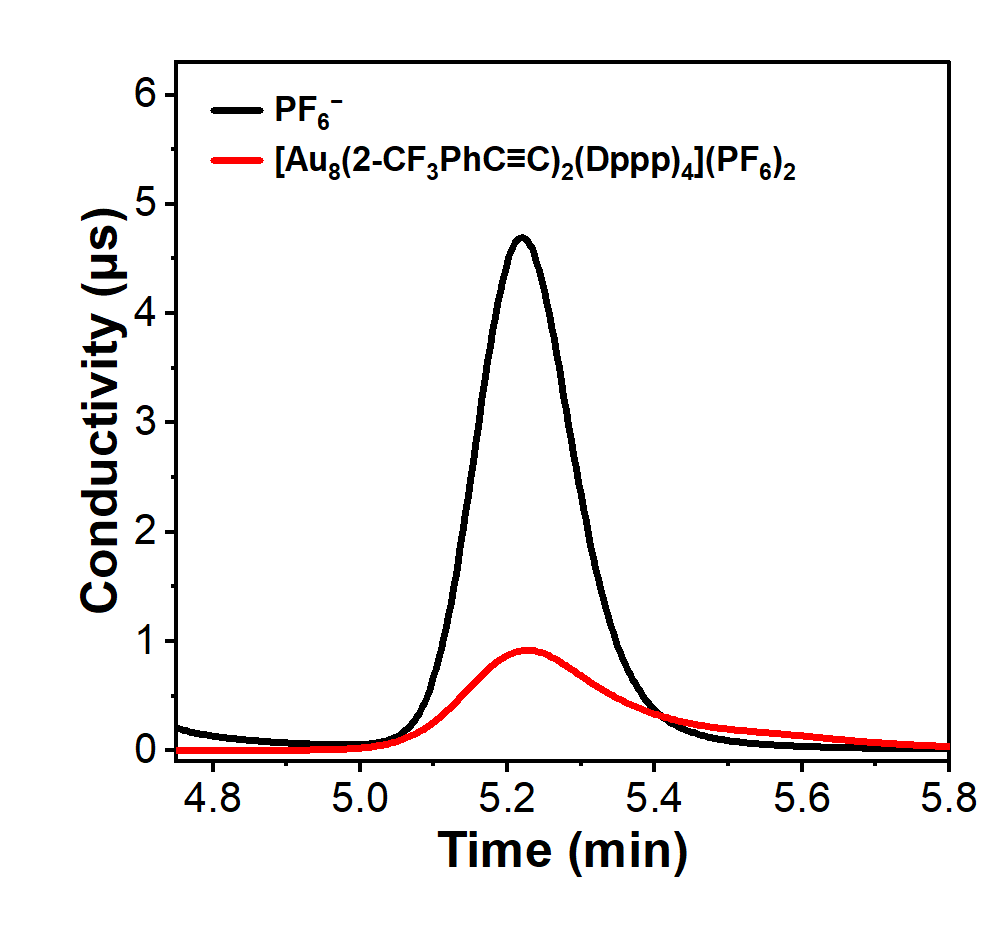


**Figure S23** The extraction of ion chromatogram of [Au₈(2-CF₃PhC≡C)₂(Dppp)₄](PF_6_)_2_ (**Au_8_-1**) (red) with PF_6_⁻ (black) as a reference.

**Part Ⅲ. Crystallographic Supplementary Information**

**Table 1**. Crystal data and structure refinement for **Au_2_(Dppe)Cl_2._**

| Empirical formula | C_26_H_24_Au_2_Cl_2_P_2_ |
| --- | --- |
| Formula weight | 863.23 |
| Temperature [K] | 149.99(10) |
| Crystal system | monoclinic |
| Space group (number) | *P*2_1_/n(14) |
| *a* [Å] | 12.7058(3) |
| *b* [Å] | 11.0936(2) |
| *c* [Å] | 18.9036(5) |
| α [°] | 90 |
| β [°] | 109.252(3) |
| γ [°] | 90 |
| Volume [Å^3^] | 2515.51(11) |
| *Z* | 4 |
| *ρ*_calc_ [gcm^−3^] | 2.279 |
| *μ* [mm^−1^] | 24.869 |
| *F*(000) | 1608 |
| Crystal size [mm^3^] | 0.5×0.3×0.3 |
| Crystal color | clear light colourless |
| Crystal shape | block |
| Radiation | Cu*K_α_* (λ=1.54184°Å) |
| 2θ range [°] | 7.40 to 151.89 (0.79°Å) |
| Index ranges | −15 ≤ h ≤ 10 −13 ≤ k ≤ 13 −22 ≤ l ≤ 23 |
| Reflections collected | 9720 |
| Independent reflections | 4652 *R*_int_ = 0.0377 *R*_sigma_ = 0.0406 |
| Completeness to θ = 67.684° | 93.4 % |
| Data / Restraints / Parameters | 4652/0/289 |
| Goodness-of-fit on *F*^2^ | 1.048 |
| Final *R* indexes [*I*≥2σ(*I*)] | *R*_1_ = 0.0354 w*R*_2_ = 0.0964 |
| Final *R* indexes [all data] | *R*_1_ = 0.0380 w*R*_2_ = 0.0980 |
| Largest peak/hole [eÅ^−3^] | 1.59/-1.28 |

**Table 2**. Crystal data and structure refinement for **Au_13._**

| Empirical formula | C_154_H_146_Au_13_Cl_3_P_10_ |
| --- | --- |
| Formula weight | 4973.34 |
| Temperature [K] | 150.15 |
| Crystal system | monoclinic |
| Space group (number) | *P*2_1_/n(14) |
| *a* [Å] | 31.3399(2) |
| *b* [Å] | 33.6647(2) |
| *c* [Å] | 33.5033(5) |
| α [°] | 90 |
| β [°] | 94.1640(10) |
| γ [°] | 90 |
| Volume [Å^3^] | 35254.3(6) |
| *Z* | 8 |
| *ρ*_calc_ [gcm^−3^] | 1.874 |
| *μ* [mm^−1^] | 21.357 |
| *F*(000) | 18384 |
| Crystal size [mm^3^] | 0.12×0.11×0.1 |
| Crystal color | clear reddish red |
| Crystal shape | block |
| Radiation | Cu*K_α_* (λ=1.54184°Å) |
| 2θ range [°] | 6.24 to 152.69 (0.79°Å) |
| Index ranges | −38 ≤ h ≤ 29 −39 ≤ k ≤ 42 −32 ≤ l ≤ 42 |
| Reflections collected | 178664 |
| Independent reflections | 69669 *R*_int_ = 0.0849 *R*_sigma_ = 0.1102 |
| Completeness to θ = 67.684° | 98.4 % |
| Data / Restraints / Parameters | 69669/2077/2789 |
| Goodness-of-fit on *F*^2^ | 1.036 |
| Final *R* indexes [*I*≥2σ(*I*)] | *R*_1_ = 0.1093 w*R*_2_ = 0.2317 |
| Final *R* indexes [all data] | *R*_1_ = 0.1922 w*R*_2_ = 0.2812 |
| Largest peak/hole [eÅ^−3^] | 3.62/-2.88 |

**Table 3**. Crystal data and structure refinement for **Au_13_-2**.

| Empirical formula | C_146_H_128_Au_13_Cl_3_F_2_P_10_ |
| --- | --- |
| Formula weight | 4897.11 |
| Temperature [K] | 149.99(10) |
| Crystal system | monoclinic |
| Space group (number) | *C*2/c(15) |
| *a* [Å] | 31.8471(3) |
| *b* [Å] | 31.8296(2) |
| *c* [Å] | 34.6802(3) |
| α [°] | 90 |
| β [°] | 115.4870(10) |
| γ [°] | 90 |
| Volume [Å^3^] | 31733.5(5) |
| *Z* | 8 |
| *ρ*_calc_ [gcm^−3^] | 2.050 |
| *μ* [mm^−1^] | 23.733 |
| *F*(000) | 18000 |
| Crystal size [mm^3^] | 0.3×0.2×0.2 |
| Crystal color | clear reddish red |
| Crystal shape | block |
| Radiation | Cu*K_α_* (λ=1.54184°Å) |
| 2θ range [°] | 5.84 to 152.56 (0.79°Å) |
| Index ranges | −38 ≤ h ≤ 38 −38 ≤ k ≤ 26 −34 ≤ l ≤ 43 |
| Reflections collected | 84625 |
| Independent reflections | 31949 *R*_int_ = 0.0338 *R*_sigma_ = 0.0371 |
| Completeness to θ = 67.684° | 99.7 % |
| Data / Restraints / Parameters | 31949/240/1552 |
| Goodness-of-fit on *F*^2^ | 1.044 |
| Final *R* indexes [*I*≥2σ(*I*)] | *R*_1_ = 0.0461 w*R*_2_ = 0.1129 |
| Final *R* indexes [all data] | *R*_1_ = 0.0557 w*R*_2_ = 0.1186 |
| Largest peak/hole [eÅ^−3^] | 1.93/-1.69 |

## Table 4. Crystal data and structure refinement for Au_8_-1.

| Empirical formula | C_126_H_112_Au_8_F_18_P_10_ |
| --- | --- |
| Formula weight | 3853.62 |
| Temperature [K] | 149.99(10) |
| Crystal system | monoclinic |
| Space group (number) | *P*2_1_/n(14) |
| *a* [Å] | 18.1266(3) |
| *b* [Å] | 18.4541(4) |
| *c* [Å] | 19.7170(3) |
| α [°] | 90 |
| β [°] | 99.566(2) |
| γ [°] | 90 |
| Volume [Å^3^] | 6503.8(2) |
| *Z* | 2 |
| *ρ*_calc_ [gcm^−3^] | 1.968 |
| *μ* [mm^−1^] | 18.277 |
| *F*(000) | 3624 |
| Crystal size [mm^3^] | 0.25×0.2×0.12 |
| Crystal color | clear orangish orange |
| Crystal shape | block |
| Radiation | Cu*K_α_* (λ=1.54184°Å) |
| 2θ range [°] | 8.69 to 133.20 (0.84°Å) |
| Index ranges | −19 ≤ h ≤ 21 −20 ≤ k ≤ 21 −23 ≤ l ≤ 23 |
| Reflections collected | 32453 |
| Independent reflections | 11467 *R*_int_ = 0.0400 *R*_sigma_ = 0.0413 |
| Completeness to θ = 66.600° | 99.7 % |
| Data / Restraints / Parameters | 11467/369/807 |
| Goodness-of-fit on *F*^2^ | 1.026 |
| Final *R* indexes [*I*≥2σ(*I*)] | *R*_1_ = 0.0506 w*R*_2_ = 0.1366 |
| Final *R* indexes [all data] | *R*_1_ = 0.0588 w*R*_2_ = 0.1429 |
| Largest peak/hole [eÅ^−3^] | 2.47/-1.77 |

## Table 5. Crystal data and structure refinement for Au_11_.

| Empirical formula | C_135_H_130_Au_11_Cl_3_P_10_ |
| --- | --- |
| Formula weight | 4335.09 |
| Temperature [K] | 149.99(10) |
| Crystal system | monoclinic |
| Space group (number) | *I*2/a(15) |
| *a* [Å] | 33.6261(5) |
| *b* [Å] | 26.5517(5) |
| *c* [Å] | 48.0564(12) |
| α [°] | 90 |
| β [°] | 106.595(2) |
| γ [°] | 90 |
| Volume [Å^3^] | 41119.0(15) |
| *Z* | 8 |
| *ρ*_calc_ [gcm^−3^] | 1.401 |
| *μ* [mm^−1^] | 15.674 |
| *F*(000) | 16080 |
| Crystal size [mm^3^] | 0.28×0.25×0.2 |
| Crystal color | clear pinkish pink |
| Crystal shape | block |
| Radiation | Cu*K_α_* (λ=1.54184°Å) |
| 2θ range [°] | 5.73 to 153.61 (0.79°Å) |
| Index ranges | −42 ≤ h ≤ 23 −23 ≤ k ≤ 33 −51 ≤ l ≤ 59 |
| Reflections collected | 109918 |
| Independent reflections | 41432 *R*_int_ = 0.0714 *R*_sigma_ = 0.0924 |
| Completeness to θ = 67.684° | 99.7 % |
| Data / Restraints / Parameters | 41432/771/1336 |
| Goodness-of-fit on *F*^2^ | 0.899 |
| Final *R* indexes [*I*≥2σ(*I*)] | *R*_1_ = 0.0555 w*R*_2_ = 0.1425 |
| Final *R* indexes [all data] | *R*_1_ = 0.1085 w*R*_2_ = 0.1633 |
| Largest peak/hole [eÅ^−3^] | 1.23/-1.47 |

## Table 6. Crystal data and structure refinement for Au_8_-2_._

| Empirical formula | C_124_H_122_Au_8_Cl_2_P_8_S_2_ |
| --- | --- |
| Formula weight | 3570.76 |
| Temperature [K] | 149.98(11) |
| Crystal system | monoclinic |
| Space group (number) | *P*2_1_/n(14) |
| *a* [Å] | 17.7838(3) |
| *b* [Å] | 17.3680(3) |
| *c* [Å] | 20.3772(4) |
| α [°] | 90 |
| β [°] | 101.042(2) |
| γ [°] | 90 |
| Volume [Å^3^] | 6177.4(2) |
| *Z* | 2 |
| *ρ*_calc_ [gcm^−3^] | 1.920 |
| *μ* [mm^−1^] | 19.422 |
| *F*(000) | 3368 |
| Crystal size [mm^3^] | 0.23×0.2×0.15 |
| Crystal color | clear pinkish pink |
| Crystal shape | block |
| Radiation | Cu*K_α_* (λ=1.54184°Å) |
| 2θ range [°] | 6.74 to 133.20 (0.84°Å) |
| Index ranges | −21 ≤ h ≤ 11 −10 ≤ k ≤ 20 −24 ≤ l ≤ 24 |
| Reflections collected | 29167 |
| Independent reflections | 10894 *R*_int_ = 0.0663 *R*_sigma_ = 0.0639 |
| Completeness to θ = 66.599° | 99.8 % |
| Data / Restraints / Parameters | 10894/556/649 |
| Goodness-of-fit on *F*^2^ | 1.019 |
| Final *R* indexes [*I*≥2σ(*I*)] | *R*_1_ = 0.0636 w*R*_2_ = 0.1672 |
| Final *R* indexes [all data] | *R*_1_ = 0.0763 w*R*_2_ = 0.1797 |
| Largest peak/hole [eÅ^−3^] | 5.84/-3.61 |

**Part Ⅳ. References**

[1] CrysAlis^Pro^ Version 1.171.35.19. Agilent Technologies Inc. Santa Clara, CA, USA, **2011**.

[2] G. M. Sheldrick, *Acta*. *Crystallogr*. *Sect. Chem*., **2015**, *71*, 3-8.

[3] O. V. Dolomanov, L. J. Bourhis, R. J. Gildea, J. A. Howard, H. Puschmann, *J. Appl. Crystallogr*, **2009**, *42*, 339-341.
